# CXCL6 is an important paracrine factor in the pro-angiogenic human cardiac progenitor-like cell secretome

**DOI:** 10.1038/s41598-017-11976-6

**Published:** 2017-10-02

**Authors:** José Luis Torán, Susana Aguilar, Juan Antonio López, Carlos Torroja, Juan Antonio Quintana, Cesar Santiago, José Luis Abad, Patricia Gomes-Alves, Andrés Gonzalez, Juan Antonio Bernal, Luis Jesús Jiménez-Borreguero, Paula Marques Alves, Luis R-Borlado, Jesús Vázquez, Antonio Bernad

**Affiliations:** 1 Department of Immunology and Oncology, Centro Nacional de Biotecnología (CNB-CSIC), Campus Universidad Autónoma de Madrid, 28049 Madrid, Spain; 2 Cardiovascular Development and Repair Department, Spanish National Cardiovascular Research Center (CNIC), Melchor Fernández Almagro 3, 28029 Madrid, Spain; 3 Cardiovascular Proteomics Laboratory, Spanish National Cardiovascular Research Center (CNIC), Melchor Fernaández Almagro 3, 28029 Madrid, Spain; 4 Bioinformatics Unit, Spanish National Cardiovascular Research Center (CNIC), Melchor Fernández Almagro 3, 28029 Madrid, Spain; 5 Cell and Developmental Biology, Spanish National Cardiovascular Research Center (CNIC), Melchor Fernández Almagro 3, 28029 Madrid, Spain; 6 Department of Macromolecular Structures, Centro Nacional de Biotecnología (CNB-CSIC), Campus Universidad Autónoma de Madrid, 28049 Madrid, Spain; 7 Coretherapix SLU, Santiago Grisolia 2, 28769 Tres Cantos, Madrid Spain; 8 Instituto de Tecnologia Química e Biológica António Xavier, Universidade Nova de Lisboa, Av. da República, 2780-157 Oeiras, Portugal; 9 iBET, Instituto de Biologia Experimental e Tecnológica, Apartado 12, 2781-901 Oeiras, Portugal; 10 Myocardial pathophysiology, Spanish National Cardiovascular Research Center (CNIC), Melchor Fernández Almagro 3, 28029 Madrid, Spain; 11 Hospital de la Princesa, Diego de León 62, 28006 Madrid, Spain

## Abstract

Studies in recent years have established that the principal effects in cardiac cell therapy are associated with paracrine/autocrine factors. We combined several complementary techniques to define human cardiac progenitor cell (CPC) secretome constituted by 914 proteins/genes; 51% of these are associated with the exosomal compartment. To define the set of proteins specifically or highly differentially secreted by CPC, we compared human mesenchymal stem cells and dermal fibroblasts; the study defined a group of growth factors, cytokines and chemokines expressed at high to medium levels by CPC. Among them, IL-1, GROa (CXCL1), CXCL6 (GCP2) and IL-8 are examples whose expression was confirmed by most techniques used. ELISA showed that CXCL6 is significantly overexpressed in CPC conditioned medium (CM) (18- to 26-fold) and western blot confirmed expression of its receptors CXCR1 and CXCR2. Addition of anti-CXCL6 completely abolished migration in CPC-CM compared with anti-CXCR2, which promoted partial inhibition, and anti-CXCR1, which was inefficient. Anti-CXCL6 also significantly inhibited CPC CM angiogenic activity. *In vivo* evaluation also supported a relevant role for angiogenesis. Altogether, these results suggest a notable angiogenic potential in CPC-CM and identify CXCL6 as an important paracrine factor for CPC that signals mainly through CXCR2.

## Introduction

The adult mammalian heart was long considered a terminally differentiated organ with no capacity to replace aged or damaged cardiomyocytes^1^. Low but intrinsic cardiomyocyte turnover has nonetheless been demonstrated in the adult mouse heart^2,3^. An external origin of heart turnover capacity, as direct transdifferentiation or fusion events, has almost been ruled out (reviewed in ref.^4^) and current discussion focuses on the contribution of specific mature cardiomyocytes by dedifferentiation/proliferation^5^ and/or of cardiac stem/progenitor cells (CSC/CPC)^6^.

The therapeutic capacity of CSC/CPC was initially attributed to their potential to differentiate into different reparative cell types. The efficiency of transplanted CSC/CPC in differentiating into functional reparative cells in injured tissues in therapeutically relevant numbers has never been adequately documented^7^. Short survival appears to be one of the main factors, although limited functional niche availability and inefficient early vascularization could be also responsible. Recent reports suggest that a substantial part of these reparative effects are mediated not by direct differentiation of engrafted CSC/CPC, but mainly by secreted paracrine factors^8,9^. These factors are postulated to promote arteriogenesis, protect against myocardial ischemia and stimulate endogenous reparative and regenerative pathways^10^, resulting in durable benefits despite transitory survival of transplanted cells^7,11,12^. CPC have demonstrated immunoregulatory capacity^13,14^, similar to mesenchymal stem cells (MSC)^15^, and an important role is proposed for PD-L1 (programmed death-ligand 1) in the immune behavior of allogeneic CPC^14^.

This paracrine hypothesis is modifying CPC use in regenerative medicine. Rather than by using cells, repair of injured tissues could be promoted by activating/priming endogenous tissue repair with a combined selection of secreted biologics, including exosome preparations. Recent evidence suggests a promising role for exosomes and extracellular vesicles in intracardiac communication (reviewed in ref.^16^), including cardiac repair and regeneration^17^. This could help to eliminate some of the problems associated with cell-based therapy such as senescence or immune incompatibility, as well as costs and waiting time in the case of autologous indications.

Numerous reports have proposed the secretion of paracrine factors as an important mechanism for mesenchymal stem cells (MSC) reparative effects on injured tissues^18,19^. The MSC secretome has been analyzed extensively (reviewed in ref.^20^), as has its early modulation during differentiation^21^. Partial CPC-associated secretome functions are also reported^22^, but there is no systematic profiling of the secretome that would enable association of the paracrine hypothesis to the spectrum of CPC-mediated therapeutic effects. Deeper comprehension of the CPC/CSC secretome and of its response to acute or diffuse chronic damage would be critical for integrated understanding of CPC therapeutic potential and damage responses.

Here we address the comparative definition of the CPC secretome, by deep proteomics analysis of several independent CPC isolates in culture conditions similar to those defined in an associated phase I/IIa clinical trial (CAREMI) in which 55 patients with large infarcts have been treated intracoronarily with allogeneic CPC^23^.

## Results

### Comparative proteomics to define the CPC secretome

To obtain a complete definition of the secreted proteome (secretome) of human CPC, we combined several complementary approaches using three independent CPC isolates (CPC1-3) and comparing with primary isolates from human bone marrow MSC, as the gold standard in cell therapy with adult stem cells^24^, and human dermal fibroblasts (HDF) as a distant reference lineage. CPC, isolated as described^14,23^, were cultured in conditions similar to those used in the associated CAREMI clinical trial^23^; all were cultured in their own optimal conditions (see Methods), using 3% O_2_, determined optimal for CPC and MSC^13,14,25^. Supplementary Fig. S1A shows a representative CPC profile compared with MSC and HDF. CPC are positive for expression of stage-specific embryonic antigen 1 (SSEA1), activated leukocyte cell adhesion molecule (CD166), Thy-1 cell surface antigen (CD90) and endoglin (CD105), and negative for hematopoietic markers as the protein tyrosine phosphatase, receptor type C (PTPRC; CD45) and the progenitor cell antigen, CD34^13,14^. Cultures were grown to 70–80% confluence, then harvested (for proteomics analyses or transcriptomics) or were FBS-deprived for 48 h, prior to harvest for conditioned medium (CM) (Supplementary Fig. S1 summarizes the scheme of analysis).

**Figure 1:**
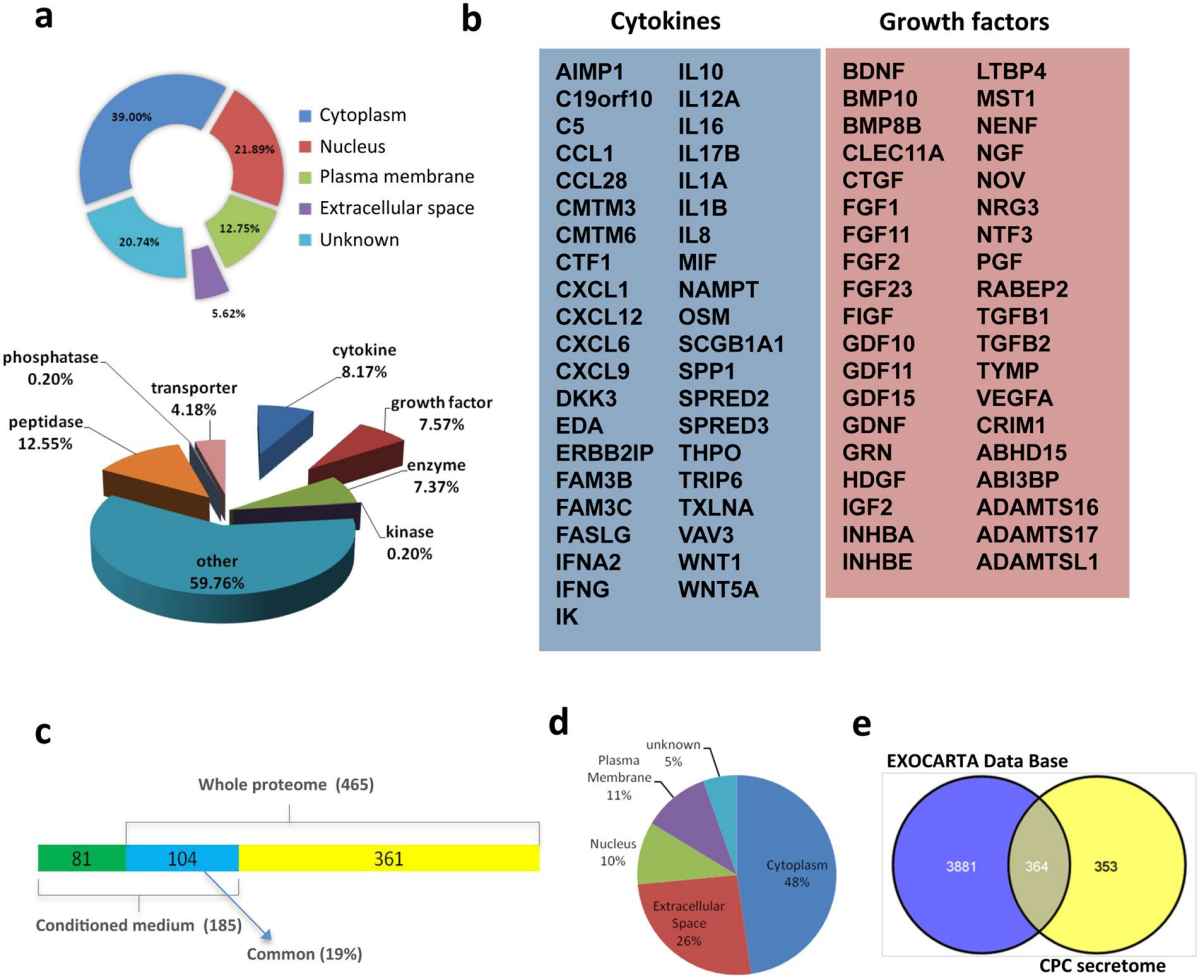
Proteomic analysis of the CPC secretome. (**a**) CPC secretome inferred from whole label-free (LF) analysis. Distribution of the CPC secretome (465 proteins) by subcellular localization (top) and by enzymatic activity (bottom). (**b**) Most relevant cytokines (left) and growth factors (right) in the CPC secretome as inferred from whole LF analysis. (**c**) Comparison of whole LF proteomics secretome and secretome obtained from CPC-conditioned medium (CM); only 19% (104; blue) of the proteins described are common. (**d**) Distribution of the CPC CM secretome by subcellular localization. (**e**) The majority of the CPC secretome (>50%) is included in the Exocarta database, indicating association with the exosome compartment.

Whole label-free (LF) proteome, obtained by LC-MS (liquid chromatography-mass spectrometry) using a LTQ-Orbitrap was reanalyzed to explore the set of proteins potentially secretable by CPC. The 465 proteins in this panel (5.62% of the proteome) (0.05% FDR, false discovery rate) were classified as secreted and/or extracellular space proteins by Ingenuity Pathway Analysis (IPA); classification by function showed 63 peptidases, 21 transporter proteins, 37 enzymes, 41 cytokines and 38 growth factors (Fig. 1a; full description in Supplementary Table S1). The main CPC-secreted cytokines and growth factors found in these experiments are listed in Fig. 1b.

In addition to the extracellular protein panel inferred from the whole LF proteomics approach, we determined the direct CPC secretome profile from CPC cell supernatants (CM, conditioned medium). CPC1 and CPC3 isolates were cultured (*n* = 3) to 60–70% confluence, FBS-deprived (48 h) and supernatants collected; after depletion of abundant proteins (DAP; see Methods); LC-MS analysis yielded 1300 proteins, with a clear increase in the numbers of less-abundant proteins. After discarding peptides derived from CPC culture control medium, we identified 754 proteins using the Uniprot database, of which IPA validated 717 (see Supplementary Fig. S2). Compared with data obtained by a whole LF proteome approach, 76.8% of proteins identified in CM were also found in the CPC total proteome set. Probable CPC contaminant proteins were found in varying amounts, including cytoplasmic, nuclear and plasma membrane proteins (summarized in Supplementary Fig. S2). Of the remaining proteins, IPA identified 185 proteins (26%) as extracellular space components (full list in Supplementary Table S2); of these, 56% (104/185) were also found by whole LF proteome analysis (Fig. 1c). Numbers and distribution of the proteins identified by the direct CPC secretome approach are summarized in Fig. 1c,d. Depletion of serum proteins notably enriched the extracellular protein fraction, enabling identification of 81 additional proteins as extracellular components of the CPC secretome (Fig. 1c). For the remaining CPC-secreted proteins, classified as cytoplasmic, membrane or nuclear, we used the EXOcarta database to analyze their potential contribution as exosome proteins (http://exocarta.org/index.html). The results showed that >50% of proteins in CPC conditioned medium (364/717) have been reported as putative exosome components (Fig. 1e), including 72 of the 185 proteins identified by depletion experiments as extracellular (39%) (Supplementary Fig. S2). IL-1A, IL-1B, IL-8, CXCL1 (GROa) and CXCL6 (PGC2) were all validated by whole LF proteomics (Fig. 1b), direct proteomics of CPC CM and ITRAQ (isobaric tags for relative and absolute quantitation; see Methods) (see Supplementary Table S3).

In summary, the combined approach used identified 546 non-redundant proteins as extracellular or secreted CPC proteins.

### Comparative validation of the CPC secretome

**Figure 2:**
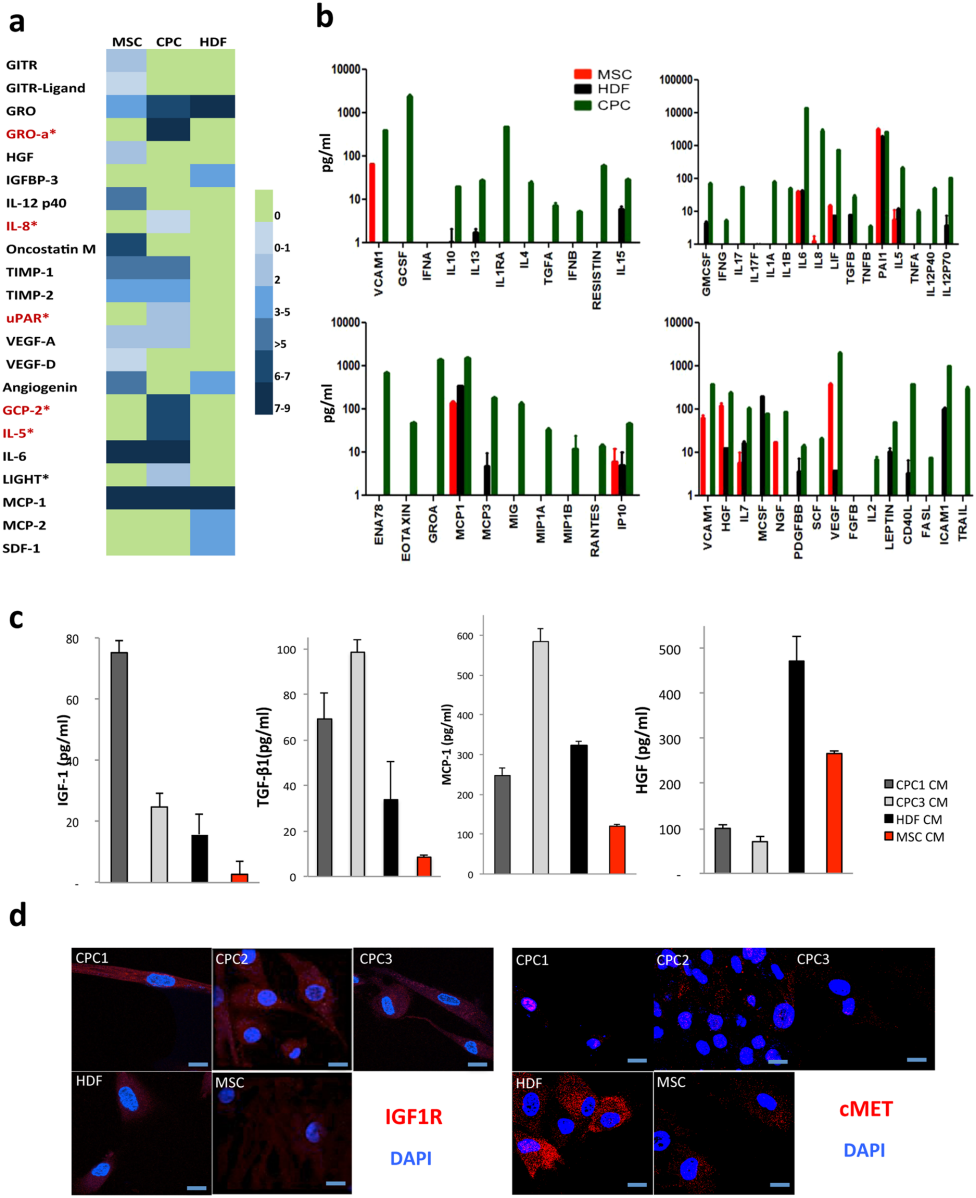
Comparative validation of the CPC secretome. (**a**) Conditioned medium from MSC, CPC and HDF were analyzed using an antibody array (122 cytokines). A color-scale of expression levels is included; proteins expressed specifically in CPC are in red. (**b**) Conditioned medium from MSC (red), HDF (black) and CPC (green) were analyzed using the Milliplex-MAP Human Cytokine/Chemokine Panel (Luminex); chemokine/cytokines are expressed in pg/ml. Duplicates were analyzed for two independent isolates from each cell lineage. (**c**) ELISA of CM from CPC1 (dark grey), CPC3 (light grey), HDF (black) and MSC (red). Duplicates were analyzed for each cell sample (*n* = 3). (**d**) Immunofluorescence analysis of IGF1R and HGFR (cMET) expression (red) in CPC1-3 compared with HDF and MSC; nuclei were DAPI-counterstained (blue). Bars, 20 μm.

CPC secretome was validated using an antibody array to determine relative protein levels and identify cytokines and growth factors secreted differentially by CPC (CPC1 and 3), HDF and MSC. Of the 122 cytokines in the antibody array, 12 were highly expressed by CPC, of which GROa (CXCL1), IL-8, uPAR, GCP-2 (CXCL6), IL-5 and LIGHT (TNFSF14) were secreted exclusively by CPC (Fig. 2a); CXCL1 and CXCL6 were expressed at very high levels. Cytokines common to CPC and MSC, but not expressed by HDF, included TIM-1, TIM-2, VEGF-A, and IL-6; only IL-6 was expressed at high levels.

For further complementary analysis, we used the Milliplex MAP Human Cytokine/Chemokine Panel (Luminex), which showed that most cytokines/chemokines included were expressed at higher levels in CPC compared with MSC > HDF. Eotaxin (CXCL5), GROa, IL-1A and IL-1B can be considered secreted specifically by CPC (Fig. 2b).

From this combined analysis, we concluded that the CPC secretome is enriched in active molecules compared with MSC/HDF secretomes. We validated GROa and IL-8 as specifically expressed by CPC compared with MSC/HDF (see Supplementary Table S3). Finally, CPC expressed IL-6 at very high levels, although the other two cell lineages also produced it (at approximately 100-fold lower concentrations).

The majority of these results were coincident in proteomics and/or genomics analysis, and a significant proportion of the proteins analyzed were preferentially expressed in CPC in comparison with MSC/HDF (Fig. 2b; summarized in Supplementary Table S3), although several proteins previously linked to CPC, such as IGF-1^26^, were not found in direct screenings (Figs 1 and 2). To confirm expression of these factors previously associated with CPC biology, including IGF-1, HGF, MCP-1, and TGF-β1^27–32^, we carried out an ELISA of CM prepared as above, which showed higher IGF-1 levels in CPC-1 and -3 than in MSC/HDF; a similar pattern was found for MCP-1 and TGF-β1 (Fig. 2c). In contrast, HGF expression was weaker in CPC than in HDF (4-fold) and MSC cells (2-fold) (Fig. 2c). IGF-1 and HGF receptor levels were also tested by immunofluorescence in all cell types. In CPC, IGF-1 receptor (IGF1R) expression was higher than in MSC and HDF, but was variable (CPC2 > CPC1 > CPC3) (Fig. 2d). HGF receptor (cMET) expression was quite variable, with the highest levels for HDF, followed by MSC and CPC (CPC2); CPC1 was negative (Fig. 2d). Our data indicate higher levels of IGF-1 secretion by CPC with higher IGF receptor levels, and suggest a feedback loop that promotes CPC survival and expansion^27^. In contrast the potential for HFG activity on CPC as an autocrine factor is low.

### Integrating proteomics and genomics

To define a more complete vision of CPC secretome, we compared CPC1-3 mRNAseq studies with those from the MSC and HDF reference cells. Of a total of 11,767 protein-coding genes in CPC, 5.8% (682) were identified as extracellular space protein using IPA; this set included 53 cytokines, 53 growth factors and 56 enzymes (see Supplementary Fig. S3 and Table S4). mRNAseq analysis of chemokines showed that *CXCL6*, *GROa*, *CXCL5* and *CCL2* were overrepresented in CPC compared to MSC/HDF. *IL-1A*, *IL-1B*, *IL-33* and *IL-8* also showed high expression in CPC compared with MSC/HDF. *IL-16* was clearly downregulated in CPC compared with MSC/HDF cells (Supplementary Figure S3).

**Figure 3:**
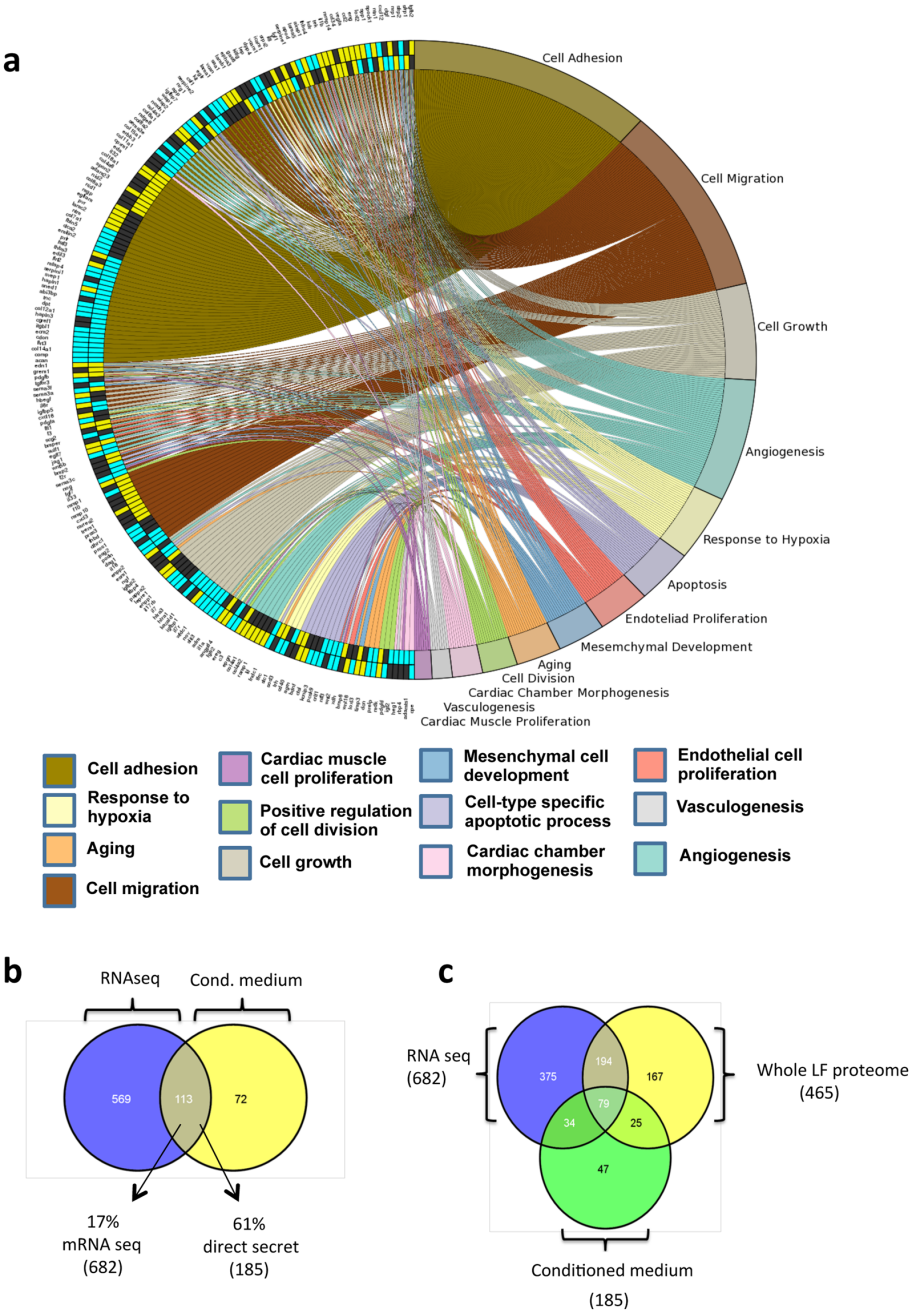
Validation of the CPC secretome inferred from the transcriptomic analysis. (**a**) Comparative GO analysis of the CPC, MSC and HDF secretomes, carried out and visualized using the GOplot application. The representation shows up- (yellow) and downregulated (blue) genes differentially expressed in CPC (p < 0.02) relative to HDF (outer circumference) or MSC (inner circumference); non-differentially expressed genes are indicated (black). Colored network connects each gene(s) with main categories analyzed. (**b**) Comparison of the CPC secretome defined by mRNAseq (682 gene/proteins) with the direct proteomics secretome obtained from CM (185 proteins). (**c**) Venn diagram of the comparison of data from mRNAseq studies (682 gene/proteins), whole LF proteomics (465 proteins) and direct secretome analysis (185 proteins); only 79 gene/proteins were found in all three data sets.

Gene ontology (GO) enrichment was analyzed in the CPC, MSC and HDF transcription-inferred secretomes with topGO and visualized using the GOplot R packages. We summarize the main categories compared, showing up- and downregulated genes differentially expressed in CPC (p < 0.05) relative to HDF and MSC; *cell adhesion*, *cell migration* and *angiogenesis* categories had the most differentially expressed genes and were most represented (Fig. 3a). In analysis by specific pathway, *cytokine pathway* and *EM structural constituted* had the largest number of upregulated genes, whereas *aging* and *BMP pathway* categories had markedly higher numbers of genes downregulated in CPC in comparison with MSC/HDF (not shown). In the main cluster associated with *cell adhesion* (Fig. 3a), some genes are overexpressed in CPC with respect to HDF/MSC (13 genes), of which *IL-32*, *ADAM23* (ADAM metallopeptidase domain 23) and *EDA* (ectodysplasin A) are associated exclusively with *cell adhesion* (Supplementary Table S5). *Angiogenesis* is one of the main categories with clear expression differences between CPC and MSC/HFD. Supplementary Figure S3 shows the main secretome functions (48 in all) related to neovascularization, endothelial cell migration and movement and to angiogenesis in general.

Using these complementary approaches, we identified a total of 914 extracellular proteins as putative components of the whole LF CPC secretome (Fig. 3b,c). From this panel, 375 proteins were detected exclusively in mRNAseq experiments, 167 were found exclusively in the whole LF CSC proteome and 47, only by the direct secretome approach on CM. Comparison of transcriptome and direct secretome data showed that 113 (16.7%) of the 682 proteins identified by mRNAseq were also identified as extracellular space proteins in the CPC minimal direct secretome (61% of 185 secreted protein identified in CM using the proteomic approach) (Fig. 3b). When compared with the whole LF proteomic set, 273 (40%) of extracellular space proteins found by mRNAseq were also identified in the CPC total proteome (Fig. 3c). In mRNAseq experiments, 55% (375) of proteins identified were detected only as coding transcripts; the remaining 307 extracellular proteins (45%) were found in direct secretome analysis, in whole LF CPC proteomics, or both (79 proteins). These intrinsic differences might be due to distinct transcription and/or translation levels, post-transcriptional regulation or differences in sensitivity and depth of the methods used^33,34^.

### Molecular and functional analysis of the CPC secretome

IPA analysis of the combined CPC secretome (Fig. 1c) is summarized in Supplementary Fig. S4; a large proportion of identified proteins (39%; 215 proteins) is involved in cell growth and proliferative signaling, similar to the number involved in cell death and survival (36%; 200) and in cell movement (27%; 154). A total of 54% (294 proteins) were classified as involved in cardiovascular development and function and 10% in immune cell trafficking.

The top 20 functional GO terms for growth factors identified in the combined CPC secretome include *regulation of angiogenesis*, *cell receptor signaling pathway*, *cell migration and motility*, *cell growth regulation*, *response to wounding* and *blood vessel morphogenesis* (p-values 8.9 10^−7^ to 2.15 10^−4^) (see Supplementary Fig. S4). This finding strongly suggests that a large fraction of CPC-released growth factors can promote remodeling via interlinked molecular signaling pathways. These results are compatible with the cell function analyses of the mRNAseq-inferred secretome (Fig. 3a).

**Figure 4:**
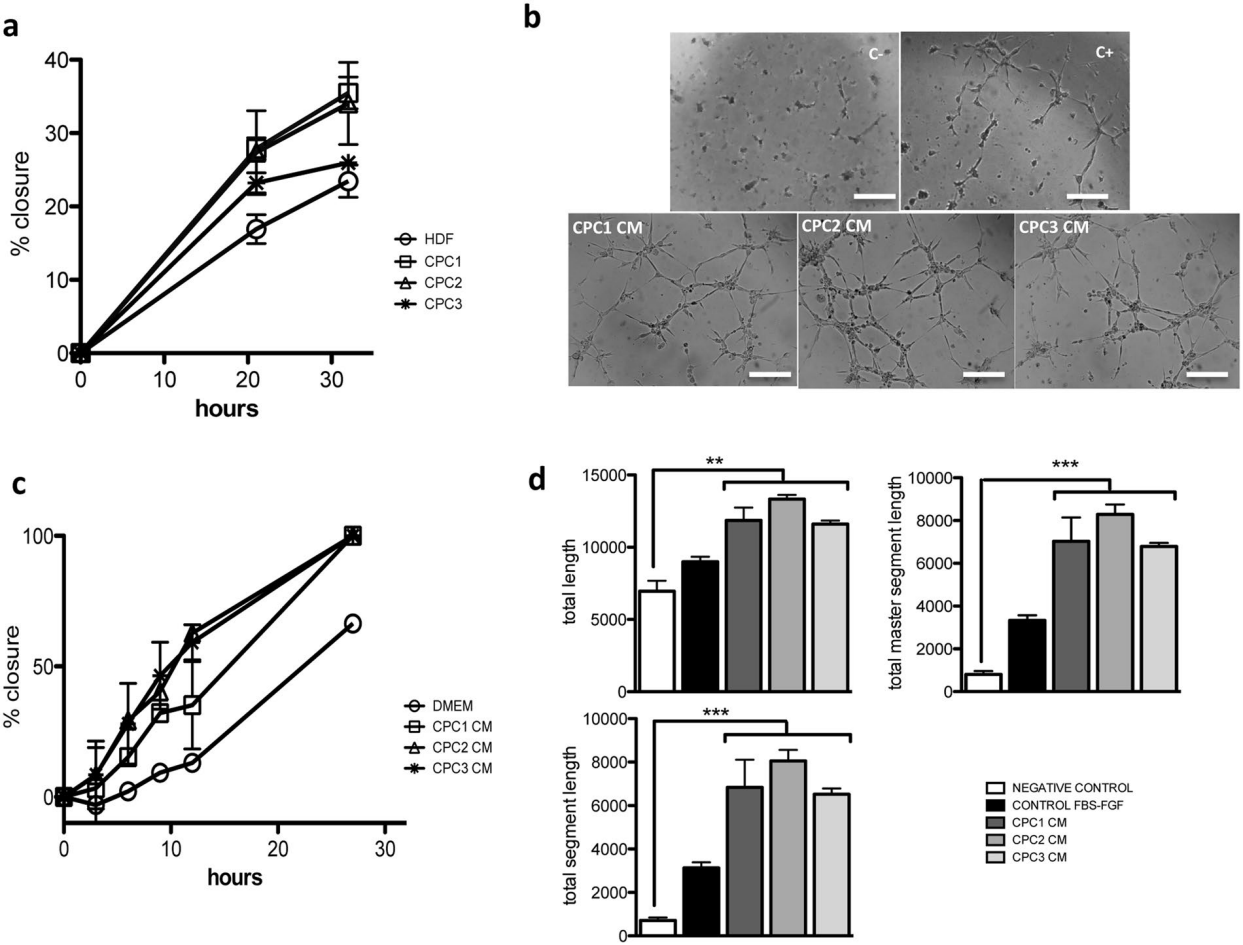
Functional evaluation of the CPC secretome. (**a**) CPC wound healing capacity. All CPC isolates (CPC1-3) showed enhanced repair capacity compared with HDF cells; expressed as the percentage of scratch closure. (**b**) HDF cells in CM from the three CPC isolates showed improved repair capacity compared with the negative control (DMEM); expressed as percentage of closure. (**c**,**d**) Angiogenic activity in CM from the three CPC isolates compared with negative (C+) and positive controls (C+), using HUVEC (see Methods); after 6 h incubation, tube formation was analyzed using ImageJ software. Bar, 500 μm. Data are expressed as mean ± SD; *n* = 3.

In an *in vitro* scratch test to analyze wound healing ability, we evaluated the capacity of CPC1-3 isolates to repair a scratch induced in subconfluent cell cultures compared with HDF cells (Fig. 4a) in serum-free medium (see Supplementary Methods). All CPC completely closed the injured area, whereas HDF showed only 60–79% healing at 30 h post-injury. We then evaluated wound-healing activity of CPC conditioned medium (CM). CPC CM were prepared from the three isolates (CPC1-3) and compared with serum-free DMEM (negative control). After scratch generation, HDF cells incubated with CPC CM showed an improved healed area compared with negative controls (35% improvement for CPC2 = CPC1 > 22% for CPC3; 30 h post-damage; *n* = 3) (Fig. 4b). This indicates a potential CPC CM capacity to participate in repair and remodeling processes via paracrine mechanisms.

These analyses led us to evaluate CPC angiogenic potential using a standard assay with HUVEC (human umbilical vein endothelial cells; see Methods), in which we compared CPC1-3 CM with negative (serum-free culture medium) and positive controls (Fig. 4c,d). Compared with negative controls, all CPC CM promoted a 2- to 2.5-fold increase in total length of tubules formed, with higher differences in the total segment length parameter (10- to 13-fold increase) (Fig. 4d; see also Supplementary Fig. S5). These results indicate that CPC secrete a combination of factors that could enhance local angiogenesis.

### Functional evaluation of CXCL6 in the CPC secretome

**Figure 5:**
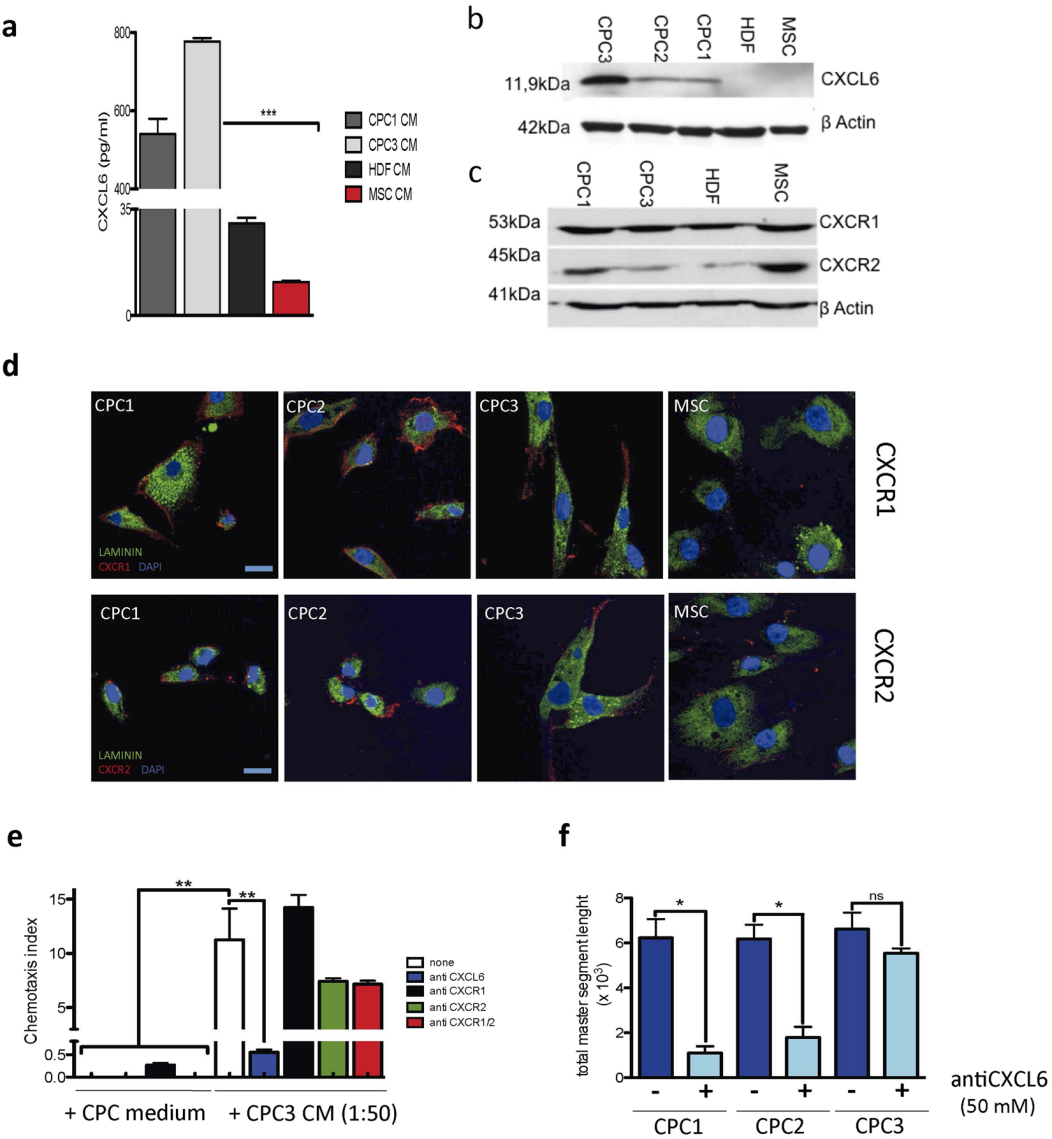
CXCL6 is an important paracrine factor in CPC CM. (**a**) ELISA evaluation of CXCL6 concentration in CPC (CPC1, CPC3), HDF and MSC CM. Data are shown as mean ± SD (*n* = 3). (**b**) Western blot analysis of CXCL6 (11.9 kDa) in CPC (CPC1-3), HDF and MSC CM; β-actin was used as loading control. (**c**) Western blot analysis of CXCR1 (53 kDa) and CXCR2 (45 kDa) in CPC (CPC1, CPC3), HDF and MSC lysates; β-actin was used as loading control. (**d**) Immunocytochemical analysis of CXCR1 and CXCR2 expression in CPC (CPC1-3) and MSC; cells were counterstained with laminin (green) and nuclei with DAPI (blue); bars, 20 μm. (**e**) Role of CXCL6 in pro-repair activity (migration) of CPC CM. Culture with CPC3 CM (1:50 dilution) induced migration of THP-1 cells in comparison with control medium. Coincubation with anti-CXCR1, anti -CXCR2 or anti -CXCL6 antibodies were tested for their ability to interfere with THP-1 cell migration. (**f**) CXCL6 in the angiogenic activity of CPC CM. HUVEC were incubated with CPC1-CPC3 CM, alone or with anti-CXCL6 antibody (50 nM). After 6 h, cultures were analyzed using ImageJ. Data expressed as mean ± SD (*n* = 3); black lines indicate p-values (***< 0.002, **< 0.02, *< 0.05; one-way analysis of variance with Bonferroni multiple comparison test).

CXCL6 (GCP-2) was one of the genes/proteins most consistently overexpressed and secreted by CPC compared with MSC and HDF cells. Transcriptomics analysis showed CXCL6 to be one of the receptor ligands with higher differential expression in CPC *vs*. MSC/HDF (11.5- and 10.5-logFC, respectively) (Supplementary Fig. 3). Quantitative proteomics also indicated differential expression (2.38-fold increase in Zq (standardized log2ratio) compared with MSC (not shown). ELISA validation showed high CXCL6 levels (550-750 pg/ml) secreted by CPC (CPC1 and CPC3), 35- and 25-fold higher than those of MSC and HDF, respectively (Fig. 5a). Western blot confirmed this high CPC expression, although with variability (CPC3 ≫ CPC2 = CPC1); CXCL6 expression was near negative in MSC/HDF (Fig. 5b). We also analyzed expression of the two known CXCL6 receptors, CXCR1 and CXCR2. Results from western blot (Fig. 5c) and immunofluorescence (Fig. 5d) indicated that both were expressed in all cell types, at variable levels. In CPC, CXCR1 levels are higher than those of CXCR2, and levels are similar to MSC (Fig. 5c,d).

For preliminary validation of the functional role of CXCL6 in CPC biology, we analyzed its involvement in migration using THP1 cells (see Supplementary Fig. S5). Recombinant CXCL6 (10 nM) stimulated migration above basal levels in transwell analysis (Supplementary Fig. S5). CM from the different CPC isolates (diluted 1:2 or 1:3) induced potent THP1 cell migration (CPC3 = CPC2 ≫ CPC1). After titration of anti-CXCL6 (Supplementary Fig. S5), anti-CXCR1 and anti- CXCR2 antibodies (Supplementary Fig. S5), we compared the migration index of THP1 exposed to from CPC3 CM (1:50) with THP-1 in the same medium co-incubated with anti-CXCL6, anti-CXCR1 and/or anti-CXCR2 antibodies. Anti-CXCR1 had no effect on migration compared with negative controls, whereas anti-CXCR2 alone provoked a 50% reduction in migration. Incubation with anti-CXCL6 almost abolished (<25-fold) THP1 cell migration capacity (Fig. 5e). As CPC showed a poor response to CXCL6 at low concentrations compared with MSC (Supplementary Fig. S5), these results suggest that CXCL6 does not have a predominant autocrine role, but is an important chemotactic factor in the CPC secretome. The HUVEC assay also confirmed CXCL6 as a relevant angiogenic factor in CPC CM. Addition of anti-CXCL6 antibody to CPC1 and CPC2 CM inhibited the potential of CM to promote angiogenic structures, expressed as total segment length (88 and 65%, respectively) (Fig. 5f). In contrast, CPC-3 CM was insensitive to anti-CXCL6 antibody (Fig. 5f), possibly because these cells produce much more CXCL6 (Fig. 5b), which might not be blocked by the anti-CXCL6 antibody.

### *In vivo* evaluation of CXCL6 function in the CPC secretome

**Figure 6:**
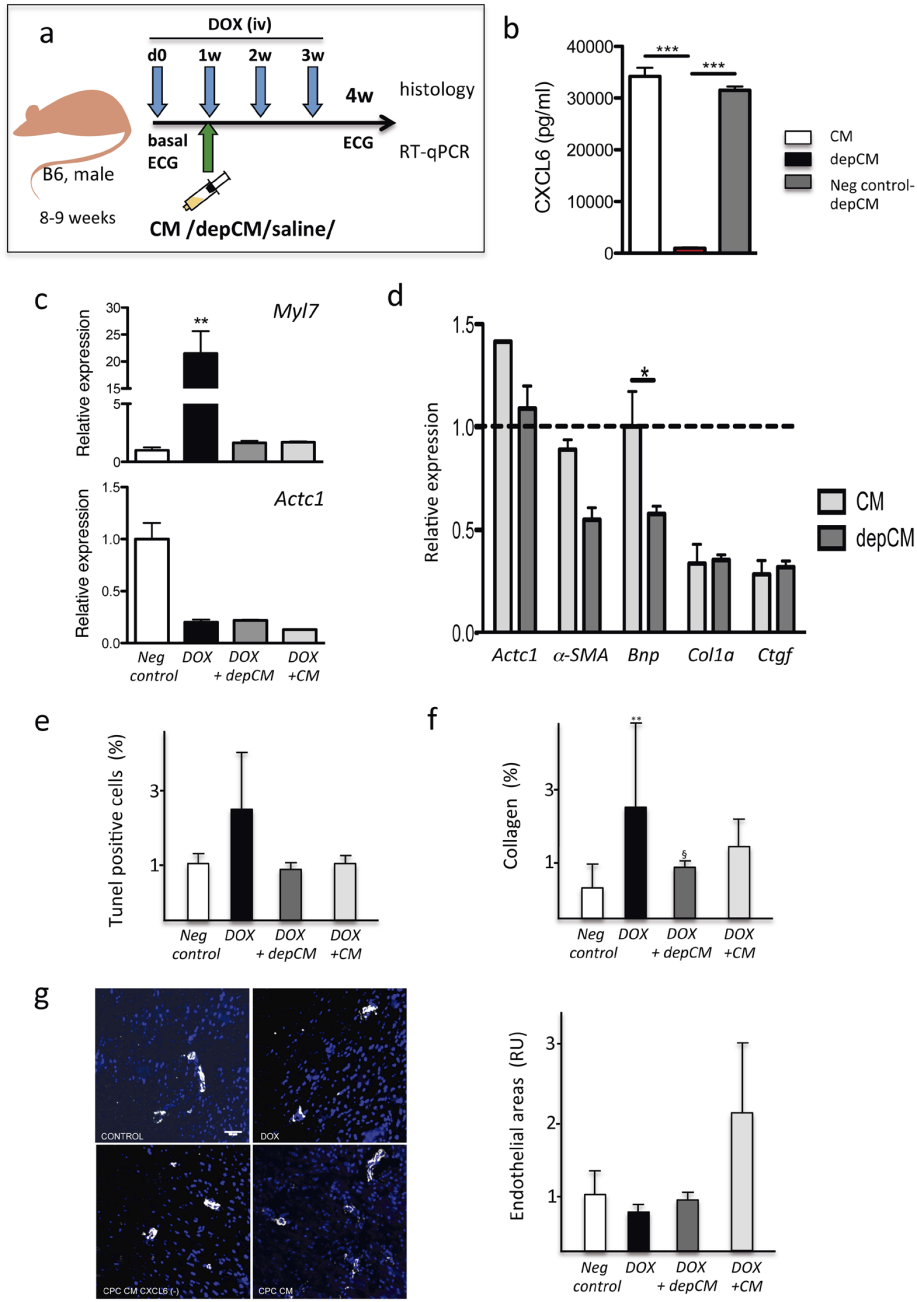
*In vivo* evaluation of CXCL6 function in a doxorubicin-induced cardiac toxicity model. (**a**) Experimental scheme. Full CPC-conditioned medium (CM; from isolate CPC3) was compared with CXCL6-depleted conditioned medium (depCM) for their capacity to counteract DOX toxicity (at 5 mg/Kg). Both CM were injected into the pericardial cavity 6 days after the first DOX dose and compared with mice that received vehicle alone (saline). Four weeks after the initial DOX dose, mice were sacrificed, the heart isolated and analyzed by RT-qPCR or in cardiac sections. Drawings design by Vicente Díaz. (**b**) ELISA quantification of CXCL6 in CM, depCM and CXCL6 bound to agarose beads. (**c**,**d**) All mouse groups were analyzed by RT-qPCR for cardiac expression of myosin light chain 7 (*Myl7*) and cardiac muscle alpha actin (*Actc1*) (**c**), and for *Des*, *Bnp*, *Casp9*, *Col1a*, *Ctgf* and *Actc1*, which are compared with values for mice treated with DOX alone (**d**). (**e**) TUNEL evaluation of apoptosis levels in cardiac sections from all mouse groups relative to the negative control. (**f**) Comparative estimation of collagen I deposition in cardiac sections, after Sirius Red staining. (**g**) Comparative quantification of angiogenic areas by staining cardiac sections from all groups with anti-CD146 and anti-αSMA: left, representative images scale bar 50 μm; right, quantification in relative units (RU). Data expressed as mean ± SD; black lines indicate p-values (***< 0.002, **< 0.02, *< 0.05; one-way analysis of variance with Bonferroni multiple comparison test).

To evaluate the *in vivo* role of CXCL6, we used a cardiac toxicity model^35,36^ induced by chronic doxorubicin (DOX) administration. Mice (B6; 8–9 w) received weekly DOX injections (5 mg/kg) and were analyzed 4 w after the first dose (Fig. 6a). Using this model, we compared treatment with full CPC-conditioned (CM; *n* = 6) or CXCL6-depleted CPC-conditioned medium (depCM; *n* = 6). depCM was prepared (see Supp Methods) and the final fractions were confirmed to have reduced (>95%) the CXCL6 content (Fig. 6b) compared to equivalent control-manipulated CM. CM preparations were injected 6 days after the first DOX dose (d0) and 1 day before the second dose (1w) into the pericardial space, essentially as described^37^; mice were sacrificed at 4 w after initiation of treatment.

RT-qPCR analysis showed that some genes such as myosin light chain 7 (*Myl7*) showed greatly increased levels (21-fold) in mice treated with DOX alone; both CM- and depCM-treated groups showed low levels similar to controls (Fig. 6c). In contrast, cardiac muscle alpha actin (*Actc1*) levels were similar in DOX, CM- and depCM-treated groups, all lower than negative controls (Fig. 6c). The results indicated that, compared with the group treated only with DOX, *Bnp*, *Des*, *and αSMA* expression was low in depCM-treated compared with CM-treated mice (Fig. 6d). No notable differences were found for *Myl7*, *Actc1* (Fig. 6c), *Col1a and Ctgf* (Fig. 6d) and many other markers (not shown).

TUNEL analysis showed that whereas mice treated with DOX alone showed a 2.5-fold increase in TUNEL^+^ cells, in CM- and depCM-treated groups, levels of TUNEL+ cells were similar to the negative control group (Fig. 6e). This result suggests that CPC-CM administration reduces DOX-promoted apoptosis, although CXCL6 appears to have no relevant role. Analysis of global free radicals by DHE staining (not shown) and fibrosis by staining with Sirius Red (Fig. 6f) yielded a similar result.

Evaluation of angiogenesis in cardiac sections by anti-CD146 and anti-αSMA staining showed a clear increase (≈2-fold) in small vessels in the CM-treated compared with the depCM-treated group (Fig. 6g). This finding coincided with the *in vitro* results; other CXCL6 functions might be masked by the rich nature of the CPC-conditioned medium.

In conclusion, using the DOX-induced cardiac toxicity model, we demonstrated that CXCL6, although it has no essential function in mediating apoptosis or fibrosis reduction appears to have a relevant role in promoting angiogenesis, probably in combination with other proteins included in CPC-conditioned medium.

## Discussion

Identification and validation of effective secreted modulators and cardioprotective proteins is currently an area of intense study. Using several complementary approaches, we analyzed the secretome of three independent CPC isolates (CPC1-3) in parallel with MSC and HDF cells, to define specific or most preferentially expressed factors. We identified 914 extracellular genes/proteins as components of the CPC secretome. Of these, 375 proteins were only detected by mRNAseq, 47 were exclusive to the direct secretome approach, and 167 were identified by whole LF CPC proteomics. Transcriptomic analysis of extracellular space proteins showed that only 16.7% (113 genes) of all genes detected in mRNAseq studies were identified by the direct secretome study. These differences are probably related to distinct transcription/translation levels, post-transcriptional regulation, or differences in sensitivity and/or precision of the methods used. Based on their own data and other similar results, Peshkin *et al*. recently proposed a non-linear model of protein dynamics of interest for proteins expressed at low levels (<10 nM), which states that a higher mRNA/protein ratio leads to better agreement between levels of mRNA and protein dynamics^34^.

Our functional analysis of CPC secretome proteins suggested a strong link with angiogenesis. We confirmed that CPC produce and secrete a combination of factors with wound healing and angiogenic activities, which demonstrates their potential for a role in repair and remodeling processes through paracrine mechanisms. Further detailed analysis and functional validation of the CPC secretome could help to define additional factors that could be used as complementary or alternative soluble paracrine or autocrine factors for human cardiac stem cell-based therapies. In a previous comparative secretome study, Stastna *et al*. identified 15 differentially expressed paracrine/autocrine factors in rat CSC compared with cultured neonatal rat ventricular myocytes^22^. Some of the proteins detected such as connective tissue growth factor (CTGF), atrial natriuretic factor (ANP) and protein interleukin-1 receptor-like 1 (ST2), were shown to reduce rat CSC proliferation. In our study, we found only CTGF and SPARC (secreted protein, acidic, cysteine-rich; osteonectin) overexpression in CPC in comparison with MSC/HDF cells.

With results derived from clinical studies using MSC, our findings here provide a footing for new approaches to cardiac therapy, as CPC/CSC and their probable associated paracrine effects have provided superior recovery results to those obtained with MSC (reviewed in refs^12,38,39^).

In this study we have demonstrated that IL-1, GROa, IL-8 and CXCL6 are expressed strongly and specifically by CPC compared with MSC/HDF. Reports concerning the physiological role of IL-1 signaling are contradictory, and depend on cell or niche context. For example, prominent IL-1A/IL-1R1 signaling was shown in the subgranular zone of adolescent mice^40^, and IL-1B was found to promote neurogenesis in the murine carotid body^41^. Comparative study of ckit + CSC from explanted human decompensated hearts and control healthy donors indicated that pathological samples show clear signs of senescence, associated mostly with enhanced IL-1B secretion and reduced levels of AMP protein kinase^10^. GROa is a proinflammatory cytokine/chemokine involved in angiogenesis that signals through the G protein-coupled receptor CXCR2. In certain contexts, GROa can also act as an inhibitor of migration, as is the case in human primary airway smooth muscle cells^42^. Plasma GROa levels correlate inversely with severity of disease in an experimental murine model of myocarditis^43^. Both GROa and CXCR2 were recently implicated in human pluripotent stem cell pluripotency and proliferation^39–44^.

The cytokine IL-8, which is involved in many pathophysiological conditions^40^, is also proposed as an angiogenesis-related factor^41–45^ that signals through its specific receptors, CXCR1 and CXCR2. CPC and MSC show relative similarities in gene/protein expression profiles and biological function^13,42–47^. Based on the high CPC levels differential expression of GROa and IL-8, and the fact that these cytokines have a common receptor, CXCR2 (Fig. 5c,d), we speculate a CPC role in regulating basal inflammation in homeostasis similar to that of MSC. In addition, CPC might likewise be recruited for focus repair after damage, with an IL-8 contribution to immunoregulation and angiogenesis. Further study is needed to define the precise role of these CPC secretome factors.

High differential CXCL6 expression by CPC was verified by ELISA (500–700 pg/ml), and expression of its specific receptors was confirmed by Western blot and immunocytochemistry in all isolates tested (Fig. 5c,d) and by whole LF proteomics analysis (Supplementary Table S1). CXCL6 was initially defined as a chemotactic factor for neutrophil/granulocytes. Kim *et al*. showed that manipulation of MSC to overexpress CXCL6 promoted high levels of pro-angiogenic genes including *VEGF-A*, *IGF-1*, *HGF* and *IL-8* ^48^. No specific role for CXCL6 has been shown in unmodified MSC^48^, but it is upregulated by IFNγ concomitantly with angiogenesis promotion^49,50^. The angiogenic properties of CXCL6 have been reported^51,52^ and that it is upregulated by hypoxia^53^. Our results also indicate a CXCL6 function in the angiogenic activity of the CPC secretome. Whereas CXCL6 elicits only a minimal migration response in CPC, our data suggest that this chemokine has an essential chemotactic function in nearby cells. Because it is one of the most consistently detected and specifically expressed proteins in CPC vs. MSC/HDF comparisons (>6000-fold change in mRNAseq; 4.58-fold increase in standardized log2ratio (Zq), in proteomics analysis), we suggest that CXCL6 is an important contributor to the enhanced angiogenic secretome.

Comparison of the combined CPC secretome profile with the Exocarta database showed 292 proteins from the secretome (>50%) reported as “exosome components” (see Supplementary Fig. S3). As notable examples of this association with highly expressed proteins in human samples, IL-5 and CCL2 are found in extracellular vesicles. In contrast, IL-6, IL-8 and CXCL6, all highly expressed in CPC, have not been described in the extracellular vesicle compartment. No definitive interpretation has been proposed for this compartmentalization^54,55^. New exosome features and abilities have recently been proposed as essential mediators of bioenergetics rescue and mitophagy in cells^56,57^.

Evaluation of the therapeutic effect of CXCL6-depleted CM compared with full CPC-CM in a model of DOX-mediated cardiac toxicity suggested that subpericardial administration of CXCL6-depleted CM promoted some differential responses. Lack of effect on regulation of apoptosis, fibrosis and ROS suggest a non-predominant CXCL6 role, although they could be masked by alternative/complementary pathways contained in the rich CPC-CM, and no enough sensitivity exist in this model.

RT-qPCR analysis confirmed that treatment of DOX-treated mice with depCM led to reduced recovery for selected genes. The clearest example is desmin, a major intermediate filament protein, essential for structural integrity and function of muscle. Desmin is described as target of DOX treatment, and is phosphorylated in Thr17, which leads to myofibrillar disarray^58^. A previous study showed that DOX promotes a dose-dependent reduction in desmin-positive cardiac microvascular endothelial cells, which induces loss of mitochondrla membrane potential and upregulates VEGF-A secretion with a concomitant reduction in its receptor expression^59^.

Using CM from genetically modified MSC that overexpress CXCL6 (MSC-CXCL6), Kim *et al*. observed significantly accelerated migration and proliferation of fibroblastic cells, as well as that MSC-CXCL6 differentiated spontaneously to endothelial-like cells^48^. Our results for administration of CPC-CM also confirmed an outcome that favored angiogenesis. CXCL6 thus appears to be a relevant factor in the CPC secretome, with a central function for the recovery of affected vessels, by acting at least on cardiac mesenchymal cells.

## Methods

### Animals

C57BL/6 mice (8- to 12-weeks-old) were provided by Charles River Laboratories Inc. Animal studies were approved by the CNB-CSIC Ethics Committee and by the Division of Animal Protection of the Comunidad de Madrid (PA 56/11, PROEX 048/16). All animal procedures conformed to EU Directive 2010/63EU and Recommendation 2007/526/EC regarding the protection of animals used for experimental and other scientific purposes, enforced in Spanish law under Real Decreto 1201/2005. Animals were maintained and handled according to the recommendations of the CNIC and CNB-CSIC institutional Ethics Committee.

### Cells and culture conditions

Human cardiac progenitor cells (CPC) were purified from three human myocardial samples by c-kit immunoselection as described^14^, expanded for full characterization (Supplementary Fig. S1)^13^ and cryopreserved. Procedures, were approved by the hospital ethical committees (H. 12 de Octubre and H. U. Gregorio Marañón, Madrid, Spain) with the corresponding patient informed consents. All methods were carried out in accordance with relevant guidelines and regulations (R.D. 9/2014 and Orden SSI/2057/2014, which transpose the European Comission Directive 2012/39/UE). CPC were maintained and expanded, as indicated (see Supplementary Methods), essentially in the same growth conditions used for the CAREMI clinical trial (EudraCT 2013-001358-81). Human bone marrow-derived mesenchymal stem cells (MSC) were obtained from the Inbiobank Stem Cell Bank (www.inbiobank.org), under the specific regulations (R.D. 1301/2006). Human foreskin fibroblasts (HDF) were purchased from American Type Culture Collection (ATCC, Manassas, VA; CRL-2097). MSC and HDF were maintained and expanded (see Supplementary Methods). All experiments were performed using cells that had undergone no more than 8 passages and were maintained in a 3% O_2_/5%CO_2_ atmosphere. The THP-1, human monocytic cell line, derived from an acute monocytic leukemia, was used for migration assays and was cultured as described (see Supplementary Methods). Human umbilical vein endothelial cells (HUVEC) were a gift from Dr. Santos Mañes (CNB-CSIC, Madrid) and were obtained from umbilical cords at term, essentially as described^60^, and purchased (cc-2517) from Lonza.

### Label-free proteomics analysis

Cells from CPC isolates CPC1-3 were used. A working cell bank was prepared (from P4 for CPC2 and P5 for CPC1 and 3) and cells were expanded to P7 and P8, respectively. After several washes in PBS, pellets (5–8 × 10^7^ cells) were collected and aliquoted. Proteins were digested using the in-gel digestion protocol as described, with some modifications (see Supplementary Methods). For direct proteomics analysis of conditioned medium (CM), cells were plated (5 × 10^3^ cells/cm^2^) in a 100 mm dish and cultured to 70–80% confluence. Cells were washed three times with serum-free medium and incubated in serum-free medium (48 h). Culture supernatants were concentrated by lyophilization filtered through a 0.22 μm filter, quantified and stored at −80 °C. Samples (200 μL) were applied to ProteoMiner columns (BioRad) to deplete abundant proteins; after washing, individual fractions (E1-E3) were collected and analyzed (20 μg total protein); CPC culture medium was processed in parallel. All proteomics data derived from this study are deposited in Peptide Atlas (http://www.peptideatlas.org/repository/) and are accessible through the accession number PASS00827.

### RNAseq analysis

RNA was isolated from CPC (CPC1-3), MSC (19, 33, 44) and HDF (F1, F2, F3) as described^13^. RNAseq libraries were obtained using the TruSeq RNA Sample Preparation v2 Kit (Illumina). Libraries were sequenced using 75 bp single-end strategy and processed as described^61^.

### Cytokine/chemokine quantification

For quantification, we used a cytokine antibody array, Luminex platform and ELISA, when indicated; for conditions, see Supplementary Methods.

### Migration and wound healing assays

For migration assays with THP-1, CPC and MSC, we used 6.5 mm polycarbonate transwells with a 5 μm-pore membrane (Corning, Corning, NY), in conditions as described (see Supplementary Methods). Wound healing assays were carried using standard conditions (Supplementary Methods).

### Angiogenic assay

Human umbilical vein endothelial cells (HUVEC) were seeded at 5 × 10^4^ cells/cm^2^ on Matrigel (356234, BD Biosciences, San Jose, CA). Cells were incubated in CPC supernatants, and serum-free medium 199 (Biowest; Nuaille, France) or medium 199 supplemented with 2% fetal bovine serum (FBS) + 3 ng/ml FGF or 5 ng/ml VEGF (6 h). Tube formation was measured using ImageJ software (NIH).

### Western blot

Cells were harvested in RIPA (radioimmunoprecipitation assay) lysis buffer, and equal amounts of lysate were separated by 10% SDS-PAGE. Proteins were transferred to PVDF membranes using the iBlot Dry Blotting System (Invitrogen, Carlsbad, CA). After incubation with primary and secondary antibodies, signals were developed using an ECL kit (GE Healthcare, Uppsala, Sweden). Supplementary Table S6 includes the list of all primary and secondary antibodies used.

### Immunofluorescence

Cells were fixed in 4% PFA, blocked with blocking buffer (PBS with 10% FBS; 30 min, room temperature), permeabilized with 0.1% Triton-X100 (5 min, room temperature), and then incubated with primary antibodies (overnight, 4 °C). After washing, cells were incubated with an appropriate secondary antibody (1 h, room temperature), washed cells were mounted in Prolong DAPI mounting medium (Invitrogen), and analyzed under a fluorescent or confocal microscope. Antibodies are summarized in Supplementary Table S6.

### Statistics

Assays were performed three times and data expressed as mean ± SD; black lines summarize p-values (***< 0.002, **< 0.02, *< 0.05) for CPC vs. HDF or MSC (one-way analysis of variance followed by the Bonferroni correction for multiple comparison).

### Data Availability

All transcriptomic data derived from this study will be deposited in Gene Expression Omnibus (*GEO*) database repository (www.ncbi.nlm.nih.gov/geo/),and are accesible through the accession number GSE84070. The rest of datasets generated during and/or analysed during the current study are available from the corresponding author on reasonable request.

### Accession codes to public repositories

All proteomics data derived from this study are deposited in Peptide Atlas (http://www.peptideatlas.org/repository/) and are accessible through the accession code PASS00827. All transcriptomic data are deposited in the Gene Expression Omnibus (GEO) database (http://www.ncbi.nlm.nih.gov/geo/query/), and are accessible through the accession number PASS00827.

## Electronic supplementary material

Supplementary Information

Table S1

Table S2

Table S4

Table S5

## References

[1] MacLellan WR, Schneider MD (2000). Genetic dissection of cardiac growth control pathways. Annu. Rev. Physiol..

[2] Laflamme MA, Murry CE (2005). Regenerating the heart. Nat. Biotechnol..

[3] Paige SL (2015). Molecular Regulation of Cardiomyocyte Differentiation. Circ. Res..

[4] Valiente-Alandi (2015). Cardiac Bmi1(+) cells contribute to myocardial renewal in the murine adult heart. Stem Cell. Res. Ther..

[5] Senyo (2013). Mammalian heart renewal by pre-existing cardiomyocytes. Nature.

[6] Senyo SE, Lee RT, Kühn B (2014). Cardiac regeneration based on mechanisms of cardiomyocyte proliferation and differentiation. Stem Cell Res..

[7] Hong (2014). c-kit+ Cardiac stem cells alleviate post-myocardial infarction left ventricular dysfunction despite poor engraftment and negligible retention in the recipient heart. PLoS One.

[8] Wang L, Gu H, Turrentine M, Wang M (2014). Estradiol treatment promotes cardiac stem cell (CSC)-derived growth factors, thus improving CSC-mediated cardioprotection after acute ischemia/reperfusion. Surgery.

[9] Izarra (2014). miR-133a enhances the protective capacity of cardiac progenitors cells after myocardial infarction. Stem Cell Rep..

[10] Avolio (2014). *Ex vivo* molecular rejuvenation improves the therapeutic activity of senescent human cardiac stem cells in a mouse model of myocardial infarction. Stem Cells.

[11] Malliaras (2014). Stimulation of endogenous cardioblasts by exogenous cell therapy after myocardial infarction. EMBO Mol. Med..

[12] Menasché P. Stem cells for the treatment of heart failure. *Philos*. *Trans*. *R*. *Soc*. *Lond*. *B*. *Biol*. *Sci*. **370**(1680), 10.1098/rstb.2014.0373 (2015). 10.1098/rstb.2014.0373 PMC4634000 26416682

[13] Moscoso *et al*. Podocalyxin-like protein 1 is a relevant marker for human c-kit+ cardiac stem cells. *J*. *Tissue Eng*. *Regen*. *Med*. **10**, 10.1002/term.1795 (2013). 10.1002/term.1795 23897803

[14] Lauden (2013). Allogenicity of human cardiac stem/progenitor cells orchestrated by programmed death ligand 1. Circ. Res..

[15] English K (2103). Mechanisms of mesenchymal stromal cell immunomodulation. Immunol. Cell Biol..

[16] Giricz Z, Varga ZV, Baranyai T (2014). Cardioprotection by remote ischemic preconditioning of the rat heart is mediated by extracellular vesicles. J. Mol. Cell Cardiol..

[17] Ibrahim AG, Cheng K, Marbán E (2014). Exosomes as critical agents of cardiac regeneration triggered by cell therapy. Stem Cell Rep..

[18] Boomsma RA, Geenen DL (2012). Mesenchymal stem cells secrete multiple cytokines that promote angiogenesis and have contrasting effects on chemotaxis and apoptosis. PLoS One.

[19] Hung (2007). Angiogenic effects of human multipotent stromal cell conditioned medium activate the PI3K-Akt pathway in hypoxic endothelial cells to inhibit apoptosis, increase survival, and stimulate angiogenesis. Stem Cells.

[20] Ranganath (2012). Harnessing the mesenchymal stem cell secretome for the treatment of cardiovascular disease. Cell Stem Cell.

[21] Rocha (2014). Secretome analysis of human mesenchymal stem cells undergoing chondrogenic differentiation. J. Proteome Res..

[22] Stastna (2010). Identification and functionality of proteomes secreted by rat cardiac stem cells and neonatal cardiomyocytes. Proteomics.

[23] Sanz-Ruiz *et al*. Rationale and design of the first-in-man clinical trial to evaluate the safety and efficacy of intracoronary infusion of allogeneic human *CAR*diac st*E*m cells in patients with acute *M*yocardial *I*nfarction and left ventricular dysfunction: the randomized multicenter double-blind controlled CAREMI trial. *Circ Res*. 2017, in press. 10.1161/CIRCRESAHA.117.310651 28533209

[24] Nancarrow-Lei, R., Mafi,. P, Mafi, R. & Khan, W. A systemic review of the sources of adult mesenchymal stem cells and their suitability in musculoskeletal applications. *Curr Stem Cell Res Ther*. 10.2174/1574888X12666170608124303 (2017).

[25] Boukouaci (2014). Natural killer cell crosstalk with allogeneic human cardiac-derived stem/progenitor cells controls persistence. Cardiovasc. Res..

[26] Li (2011). Expansion of human cardiac stem cells in physiological oxygen improves cell production efficiency and potency for myocardial repair. Cardiovasc Res..

[27] Torella (2004). Cardiac stem cell and myocyte aging, heart failure, and insulin-like growth factor-1 overexpression. Circ. Res..

[28] Johnson AM, Kartha CC (2014). Proliferation of murine c-kit(pos) cardiac stem cells stimulated with IGF-1 is associated with Akt-1 mediated phosphorylation and nuclear export of FoxO3a and its effect on downstream cell cycle regulators. Growth Factors.

[29] Ellison (2011). Endogenous cardiac stem cell activation by insulin-like growth factor-1/hepatocyte growth factor intracoronary injection fosters survival and regeneration of the infarcted pig heart. J. Am. Coll. Cardiol..

[30] Boucek (2015). A. *Ex vivo* paracrine properties of cardiac tissue: Effects of chronic heart failure. J. Heart Lung Transplant..

[31] Chen (2013). Human pericytes for ischemic heart repair. Stem Cells.

[32] Kawaguchi N (2012). Stem cells for cardiac regeneration and possible roles of the transforming growth factor-β superfamily. Biomol. Concepts.

[33] Vogel C, Marcotte EM (2012). Insights into the regulation of protein abundance from proteomic and transcriptomic analyses. Nat. Rev. Genet..

[34] Peshkin (2015). On the Relationship of Protein and mRNA Dynamics in Vertebrate Embryonic Development. Dev. Cell.

[35] Li (2016). Doxorubicin blocks cardiomyocyte autophagic flux by inhibiting lysosome acidification. Circulation.

[36] Zhang (2015). Potent paracrine effects of human induced pluripotent stem cell derived mesenchymal stem cells attenuate doxorubicin-induced cardiomyopathy. Sci Rep..

[37] Laakmann (2013). Minimally invasive closed-chest ultrasound-guided substance delivery into the pericardial space in mice. Naunyn Schmiedebergs Arch Pharmacol..

[38] Khanabdali *et al*. Harnessing the secretome of cardiac stem cells as therapy for ischemic heart disease. *Biochem*. *Pharmacol*. S0006-2952(16)00111-8 (2016). 10.1016/j.bcp.2016.02.012 26903387

[39] Goumans MJ, Maring JA, Smits AM (2014). A straightforward guide to the basic science behind cardiovascular cell-based therapies. Heart.

[40] McPherson CA, Aoyama M, Harry GJ (2011). Interleukin (IL)-1 and IL-6 regulation of neural progenitor cell proliferation with hippocampal injury: differential regulatory pathways in the subgranular zone (SGZ) of the adolescent and mature mouse brain. Brain Behav. Immun..

[41] Xue (2015). Interleukin-1β promotes the neurogenesis of carotid bodies by stimulating the activation of ERK1/2. Respir. Physiol. Neurobiol..

[42] Al-Alwan (2014). CXCL1 inhibits airway smooth muscle cell migration through the decoy receptor Duffy antigen receptor for chemokines. J. Immunol..

[43] Bachmaier K, Toya S, Malik AB (2014). Therapeutic administration of the chemokine CXCL1/KC abrogates autoimmune inflammatory heart disease. PLoS One.

[44] Jung (2015). CXCR2 and its related ligands play a novel role in supporting the pluripotency and proliferation of human pluripotent stem cells. Stem Cells Dev..

[45] Baggiolini M (2015). CXCL8 - The First Chemokine. Front. Immunol..

[46] Guichet (2015). Notch1 stimulation induces a vascularization switch with pericyte-like cell differentiation of glioblastoma stem cells. Stem Cells.

[47] Gomes-Alves (2015). Exploring analytical proteomics platforms toward the definition of human cardiac stem cells receptome. Proteomics.

[48] Kim (2012). Mesenchymal stem cells overexpressing GCP-2 improve heart function through enhanced angiogenic properties in a myocardial infarction model. Cardiovasc. Res..

[49] Pattappa (2014). CCL5/RANTES is a key chemoattractant released by degenerative intervertebral discs in organ culture. Eur. Cell Mater..

[50] Strojny (2015). Interferon Gamma-treated Dental Pulp Stem Cells Promote Human Mesenchymal Stem Cell Migration *In Vitro*. J. Endodont..

[51] Rot A, von Andrian UH (2004). Chemokines in innate and adaptive host defense: basic chemokinese grammar for immune cells. Annu. Rev. Immunol..

[52] Strieter (1995). The functional role of the ELR motif in CXC chemokine-mediated angiogenesis. J. Biol. Chem..

[53] Tian (2014). HIF-1alpha plays a role in the chemotactic migration of hepatocarcinoma cells through the modulation of CXCL6 expression. Cell Physiol. Biochem..

[54] Baglio (2015). Human bone marrow- and adipose-mesenchymal stem cells secrete exosomes enriched in distinctive miRNA and tRNA species. Stem Cell. Res. Ther..

[55] Cervio E, Barile L, Moccetti T (2015). Exosomes for Intramyocardial Intercellular Communication. Stem Cells Int..

[56] Akyurekli (2015). A systematic review of preclinical studies on the therapeutic potential of mesenchymal stromal cell-derived microvesicles. Stem Cell Rev..

[57] Phinney (2015). Mesenchymal stem cells use extracellular vesicles to outsource mitophagy and shuttle microRNAs. Nat. Commun..

[58] Gratia (2012). Cardiac phosphoproteome reveals cell signaling events involved in doxorubicin cardiotoxicity. J Proteomics.

[59] Chiusa (2012). Cancer therapy modulates VEGF signaling and viability in adult rat cardiac microvascular endothelial cells and cardiomyocytes. J Mol Cell Cardiol..

[60] Segura (2002). Inhibition of programmed cell death impairs *in vitro* vascular-like structure formation and reduces *in vivo* angiogenesis. FASEB J..

[61] Nakazato T, Ohta T, Bono H (2013). Experimental design-based functional mining and characterization of high-throughput sequencing data in the sequence read archive. PLoS ONE.

