# Supplementary material for: CXCL6 is an important paracrine factor in the pro-angiogenic human cardiac progenitor-like cell secretome
Source: Sci Rep. 2017 Oct 2;7:12490. doi: 10.1038/s41598-017-11976-6 (PMC5624898; doi:10.1038/s41598-017-11976-6)
Supplement: Supplementary file 1 — Supplementary Information [file 41598_2017_11976_MOESM1_ESM.doc]

**CXCL6 is an important paracrine factor in the pro-angiogenic**

**human cardiac progenitor-like cell secretome**

José Luis Torán^1,2,♯^, Susana Aguilar^1,2, ♯^, Juan Antonio López^3^, Carlos Torroja^4^, Juan Antonio Quintana^2,5^, Cesar Santiago^6^, José Luis Abad^7^, Patricia Gomes-Alves^8,9^, Andrés Gonzalez^10^, Juan Antonio Bernal^10^, Luis Jesús Jiménez-Borreguero^5^, Paula Marques Alves^7,8^, Luis R-Borlado^6^, Jesús Vázquez^3^, Antonio Bernad^1,2,*^

**Running title:** *Human CPC secretome*

^1^Department of Immunology and Oncology, Centro Nacional de Biotecnología (CNB-CSIC), Campus Universidad Autónoma de Madrid, 28049 Madrid, Spain

^2^Cardiovascular Development and Repair Department, Spanish National Cardiovascular Research Center (CNIC), Melchor Fernández Almagro 3, 28029, Madrid, Spain

^3^Cardiovascular Proteomics Laboratory, Spanish National Cardiovascular Research Center (CNIC), Melchor Fernández Almagro 3, 28029, Madrid, Spain

^4^Bioinformatics Unit, Spanish National Cardiovascular Research Center (CNIC), Melchor Fernández Almagro 3, 28029, Madrid, Spain

^5^Cell and Developmental Biology, Spanish National Cardiovascular Research Center (CNIC), Melchor Fernández Almagro 3, 28029, Madrid, Spain. Hospital de la Princesa, Diego de León 62,
28006 - Madrid

^6^Department of Macromolecular Structures, Centro Nacional de Biotecnología (CNB-CSIC), Campus Universidad Autónoma de Madrid, 28049 Madrid, Spain

^7^Coretherapix SLU, Santiago Grisolia 2, 28769, Tres Cantos, Madrid, Spain

^8^Instituto de Tecnologia Química e Biológica António Xavier, Universidade Nova de Lisboa, Av. da República, 2780-157 Oeiras, Portugal;

^9^iBET, Instituto de Biologia Experimental e Tecnológica, Apartado 12, 2781-901 Oeiras, Portugal;

^10^Mycardial pathophysiology. Spanish National Cardiovascular Research Center (CNIC), Melchor Fernández Almagro 3, 28029, Madrid, Spain

^♯^Equal contribution

**(*) Corresponding author:** Antonio Bernad. Department of Immunology and Oncology, Centro Nacional de Biotecnología (CNB-CSIC), Campus Universidad Autónoma de Madrid, 28049 Madrid, Spain. E‑mail: abernad@cnb.csic.es; Telephone: +34-915855424; Fax: +34-91-585 4506

**SUPPLEMENTARY INFORMATION**

**Supplementary Methods**

**Cells and culture conditions**

Human cardiac biopsies were obtained from patients suffering from an open-chest surgery, usually for valve replacement. Starting material was obtained from the right atria appendage, which is routinely removed in order to place the cannulae for the extracorporeal circulation. Tissue samples were minced into small pieces (<1 mm3) and treated with collagenase type 2 (Worthington Biochemical Corporation, Lakewood, NJ, USA) for 3 cycles of 30 min each to obtain a cellular suspension. Cardiomyocytes were removed by centrifugation and filtration using 40 μm cell strainers. Human cardiac progenitor cells (CPC) were purified from 3 human myocardial samples after immunodepletion of CD45-positive cells by c-kit immunoselection, following manufacturer recommendations, as described (1, 2). CPC were expanded for full characterization (Supplementary Fig. S1; 3) and, subsequently, cryopreserved.) Cells were maintained in DMEM/F12 and neurobasal medium (1:1), (both from Invitrogen; Madrid, Spain), supplemented with 10% fetal bovine serum embryonic stem cell-qualified (FBS ESCq, Invitrogen), 2 mM L-glutamine (Lonza; Belgium), penicillin-streptomycin (100 U/mL and 1000 U/mL, both from Lonza), 0.5X B27 supplement, 0.5X N2 supplement, 10 ng/mL bFGF and 0.5X ITS (all from Invitrogen), 30 ng/mL IGF-II and 20 ng/mL EGF (both from Peprotech; Neuilly-sur-Seine, France). Human bone marrow-derived mesenchymal stem cells (MSC) were obtained from the Inbiobank Stem Cell Bank (www.inbiobank.org) and maintained in Dulbecco’s Modified Eagle Medium-low glucose (DMEM-low glucose; Sigma) supplemented with 10% FBS (Sigma), 2 mM glutamine (Lonza), 100 IU/ml penicillin and 1000 U/ml streptomycin (Lonza). Human foreskin fibroblasts (HDF) were purchased from the American Type Culture Collection (CRL-2097; ATCC, Manassas, VA, USA). Cells were maintained in DMEM (Sigma) supplemented with 10% FBS, 2 mM glutamine (Lonza), 100 U/ml penicillin and 1000 IU/ml streptomycin (Lonza). All experiments were performed with cells at no more than 8 passages maintained in a 3% O_2_ / 5% CO_2_ atmosphere. HUVEC cells maintained in gelatin-coated T-75 culture flasks (37ºC, 5% CO2, ≈95% humidity) using Medium 199 (Sigma) supplemented with 10%FBS (Sigma), 10mM HEPES (Lonza), 3μg/ml endothelial cell growth supplement (ECGS, Sigma), 2mM glutamine (Lonza) and penicillin (100 U/ml) /streptomycin (1000 U/ml), (both from Lonza). THP-1 cells were cultured (37ºC, 5% CO2, ≈95% humidity) in RPMI (Sigma) supplemented with 10% FBS (Sigma), 2mM glutamine (Lonza), 50μM 2-mercaptoethanol (Sigma), penicillin (100 U/ml) /streptomycin (1000 U/ml) (both from Lonza).

**Label-free proteomics analysis**

Proteins were digested using the in-gel digestion protocol as described (4). Briefly, supernatants were resolved by conventional SDS-PAGE until the front entered 3 mm into the concentrating gel. The protein band containing the whole proteome was visualized by Coomassie staining, excised, cut into cubes, subjected to reduced using 10 mM dithiothreitol (DTT) and alkylation in 50 mM iodoacetamide, and digested (overnight, 37ºC) with 60 ng/ml modified trypsin (Promega, Madison, WI, USA) at a 12:1 protein:trypsin (w/w) ratio in 50 mM ammonium bicarbonate pH 8.8, containing 10% acetonitrile. Resulting tryptic peptides were extracted by incubation in 12 mM ammonium bicarbonate pH 8.8 and followed by 0.5% trifluoroacetic acid (TFA). TFA was added to a final concentration of 1% and peptides were desalted on C18 Oasis-HLB cartridges and dried for further analysis. Tryptic peptides were dissolved in 0.1% formic acid (FA) and loaded on a liquid chromatography-mass spectrometry (LC-MS/MS) system for online desalting on C18 cartridges and further analysis by LC-MS/MS, using a C-18 reverse-phase nanocolumn (75 μm ID x 50 cm, 3 μm particle size, Acclaim PepMap 100 C18, Thermo-Fisher), in a continuous (0-30%) acetonitrile gradient consisting of B (90% acetonitrile, 0.5% formic acid), in 180 min, 30-43% in 5 min and 43-90% in 2 min. A ~200 nL/min flow rate was used to elute peptides from the reverse phase nanocolumn to an emitter nanospray needle for real time ionization and peptide fragmentation on orbital ion trap mass spectrometers (Orbitrap Elite mass spectrometer, Thermo-Fisher).

**Database searching**

For peptide identification, MS/MS spectra were searched with the SEQUEST HT algorithm implemented in Proteome Discoverer 1.4.0.29 (Thermo Scientific). For database searching at the Uniprot database containing all sequences from human genome (March 6, 2013; 70024 entries; and Mann’s lab contaminants), search parameters were selected as follows: trypsin digestion with 2 maximum missed cleavage sites, precursor and fragment mass tolerances of 800 ppm and 1.2 Da, respectively, carbamidomethyl cysteine as fixed modification and methionine oxidation as dynamic modifications. Peptide identification was validated using the probability ratio method (5) with an additional filtering for a precursor mass tolerance of 15 ppm (6), and FDR were calculated using inverted databases and a refined method to calculate false discovery rates for peptide identification using decoy databases (7). All proteomics data derived from this study are deposited in Peptide Atlas (http://www.peptideatlas.org/repository/) and are accessible through the accession number PASS00827.

**RNA-Seq analysis**

*RNA-Seq library production*. RNAseq libraries were obtained using the TruSeq RNA Sample Preparation v2 Kit (Illumina, San Diego, CA). The quality, quantity and the size distribution of the Illumina libraries were determined using the DNA-1000 Kit (Agilent Bioanalyzer). Libraries were sequenced (single-end mode, 75 bp length) on the Genome Analyzer IIx following the standard RNA sequencing protocol, with the TruSeq SBS Kit v5.  Fastq files containing reads for each library were extracted and demultiplexed using the Casava v1.8.2 pipeline. Sequenced reads were quality-controlled and pre-processed using cutadapt v1.6 (8) to remove adaptor contaminants as described previously (9). Resulting reads were aligned and gene expression quantified using RSEM v1.1.19 (10) over human reference GRCh37 and Ensembl genebuild 65. Only genes with at least 1 count per million in at least 3 samples were considered for statistical analysis. Data were then normalized and deferential expression tested using the bioconductor package EdgeR v3.0.8 (11). We considered as differentially expressed those genes with a Benjamini-Hochberg adjusted p‑value ≤0.05. For the set of differentially expressed genes, functional analysis was performed using the topGO v2.10 Bioconductor R package (12) with annotations from org.Hs.eg.db and GO.db v2.8. For functional analysis, the genes at the *extracellular space* according to its GO annotations were used. Enrichment was performed using the full list of equally localized genes as reference. Top biological processes and molecular functions were selected using the Weighted Fisher method implemented by topGO with p<0.01. To visualize relationships between genes and biological processes, a chord plot was generated using the R visualization package GOPlot (13). All transcriptomic data derived from this study are deposited in the Gene Expression Omnibus (GEO) database, and are accesible through the accession number PASS00827.

**Migration assay**

THP-1 cell migration was assayed using 6.5mm transwell polycarbonate membrane of 5μm pore (Corning, NY). THP-1 monocytes were washed twice with Gey’s balanced salt solution (GBSS; G9779, Sigma, St. Louis, MO) with 0.1% BSA and cells (250,000 cells/100 μl) were seeded into upper chambers. The CPC CM (48 h), human CXCL6/GCP-2 (333-GC, R&D Systems, Minneapolis, MN) and their respective negative controls (serum-free medium or GBSS with 0.1% BSA) were plated in lower transwell chambers. Migration was inhibited using anti-CXCL6 antibody or by blocking receptors with anti-CXCR1 and -CXCR2 antibody (R&D Systems, Minneapolis, MN). After incubation (3 h, 37ºC, 5% CO_2_), cells that migrated into the lower chamber were collected and counted in BD TruCOUNT tubes (340334, BD Pharmingen, Heidelberg, Germany).

To determine CPC or MSC cell migration using human CXCL6/GCP-2 as a positive chemotaxis factor, cells were seeded in the upper chamber of 6.5 mm transwells with 8 μm pore polycarbonate membrane insert (Corning) and incubated (CPC, 15 h; MSC, 24 h; both at 37ºC, 3% O_2_ and 5%CO_2_). Cells that migrated to the lower chamber were collected and counted in BD TruCOUNT tubes.

**Wound healing assay**

CPC and HDF cells were cultured to confluence and starved in serum-free medium (24 h). The cell monolayer was scraped with a pipette tip; CPC and HDF cells were incubated in serum-free DMEM or CPC CM (48 h). Images were acquired after scratching and migration rates were measured using ImageJ software (US National Institutes of Health, Bethesda, MD).

**Cytokine/chemokine quantification**

*Cytokine antibody array*. Cells were cultured in 6-well plates at 90% confluence and incubated in 2.5 ml/well serum-free medium (48 h). Cell culture supernatants and controls (culture medium) were incubated with array membranes (Raybio Human Cytokine Antibody Array C Series 1000, RayBiotech; Norcross, GA) according to manufacturer's instructions. The array allows the detection of 122 cytokines: angiogenin, BDNF, BLC, BMP-4, BMP-6, CK beta 8-1, CNTF, EGF, eotaxin, eotaxin-2, eotaxin-3, FGF-6, FGF-7, Flt-3 ligand, fractalkine, GCP-2, GDNF, GM-CSF, I‑309, IFN-γ, IGFBP-1, IGFBP-2, IGFBP-4, IGF-I, IL-10, IL-13, IL-15, IL-16, IL-1A, IL-1B, IL-1RA, IL-2, IL-3, IL-4, IL-5, IL-6, IL-7, leptin, LIGHT, MCP-1, MCP-2, MCP-3, MCP-4, M-CSF, MDC, MIG, MIP-1d, MIP-3A, NAP-2, NT-3, PARC, PDGF-BB, RANTES, SCF, SDF-1, TARC, TGF-β1, TGF-β 3, TNF-α, TNF-β (array IV); and Acrp30, AgRP, angiopoietin-2, amphiregulin, Axl, bFGF, b-NGF, BTC, CCL-28, CTACK, Dtk, EGF-R, ENA-78, Fas, TNFRSF6, FGF-4, FGF-9, GCSF, GITR ligand, GITR, GRO, GRO-a, HCC-4, HGF, ICAM-1, ICAM-3, IGFBP-3, IGFBP-6, IGF-I SR, IL-1 R4/ST2, IL-1 RI, IL-11, IL-12 p40, IL-12 p70, IL-17, IL-2 Ra, IL-6 R, IL-8, I-TAC, lymphotactin, MIF, MIP-1α, MIP-1β, MIP-3β, MSP-α, NT-4, osteoprotegerin, oncostatin M, PLGF, sgp130, sTNF RII, sTNF-RI, TECK, TIMP-1, TIMP-2, thrombopoietin, TRAIL R3, TRAIL R4, uPAR, VEGF-A, VEGF-D (array VI).

*Luminex*. Cell culture supernatants (48 h; in serum free medium) and controls (serum-free medium) were analyzed by Luminex MAP200 at the Human Immune Monitoring Core (HIMC), Stanford University.

*ELISA*. Cytokine concentrations (IGF-1, HGF, MCP-1, TGF-B1 and CXCL6) were measured with ELISA kits (Quantikine, R&D Systems) according to manufacturer’s recommendations.

**Flow cytometry**

CPC, MSC and HDF cells were detached with trypsin-EDTA, washed with PBS and 0.01% BSA, and incubated with primary antibody (1 h, on ice). After extensive washing, cells were incubated with fluorescent secondary antibody (30 min, on ice), washed with PBS + 0.01% BSA and analyzed on a FACS Canto 3L flow cytometer (BD Biosciences, San Jose, CA). Supplementary Table S6 includes the list of all primary and secondary antibodies used.

**CXCL6-immunodepleted CM preparation**

Mouse anti-human CXCL6 antibody (1 mg; R&D Sytems) was covalently coupled to CNBr-preactivated sepharose beads (GE Healthcare Lifesciences) following manufacturer instructions. Beads were treated with 1mM HCl for 15 mins, mixed with the antibody dissolved in coupling buffer (0.1 M NaHCO3 pH 8.3 / 0.5 M NaCl) and incubated overnight at 4ºC. Uncoupled antibody was washed out with coupling buffer and remaining active groups blocked after incubation with 1M ethanolamine pH  8.0. Beads were finally washed with 0.1 M acetic acid/sodium acetate, pH 4.0/ 0.5 M NaCl followed by a wash with 0.1 M Tris-HCl, pH 8/ 0.5 M NaCl and stored in PBS. CPC CM, obtained as previously described, was deplete by incubation at 4ºC for 90 minutes with soft shaking and agarose beads were removed by sedimentation. For quantitation, CXCL6 was removed from agarose beads with 2M glicine pH 3.0 and neutralized using 1M Tris-HCl pH 8.0. CXCL6 concentration was measured with ELISA kits (Quantikine, R&D Systems) according to manufacturer’s recommendations.

**Animal model of cardiac toxicity**

Animal studies were approved by the CNB-CSIC ethics committee and by the Division of Animal Protection of the Comunidad de Madrid (PA 56/11, PROEX 048/16). All animal procedures conformed to EU Directive 2010/63EU and Recommendation 2007/526/EC regarding the protection of animals used for experimental and other scientific purposes, enforced in Spanish law under Real Decreto 1201/2005.

The animal model of anthracycline-induced cardiomyopathy (AIC) was induced in adult mice (6–8 weeks, B6 strain) by intravenous injection of Dox (5mg/kg), once per week, during four consecutive weeks. In the negative control group, mice were injected with an equal volume of saline. Animals were randomizated and monitored by echocardiography along the whole process. These two groups (positive and negative; n=4, each) were compared with animals (n=6) treated with a single injection with full CPC-CM (100 μl) or an equivalent group (n=6) injected with CXCL6-depleted CPC-CM (100 μl) in the pericardial cavity, as previously described (14), 1 day before the second injection of DOX (Figure 6A). Four weeks weeks after the first DOX administration all mice were then sacrificed and RT-qPCR analysis and detailed studies on cryosections were performed to assess myocardial fibrosis, apoptosis, oxidative stress and on-going angiogenesis.

**Histological analysis**

All mice were sacrificed and hearts were harvested and fixed with 4% PFA and embedded in OCT, then sectioned to 10 μm slides. Histochemical detection of apoptotic cells in heart sections was determined with the TUNEL assay kit (Roche). Sirius red staining was performed according to the manufacturer’s protocols (Sigma) and total endothelial area was determined by staining with anti-CD146 and anti-αSMA. All heart sections were analyzed and quantificated using Image, as previously described (15).

**RT-qPCR analysis**

RNA was purified using the Cells-to-CT kit (Ambion, Thermo). Complementary DNA was obtained by reverse transcription with the High Capacity cDNA Reverse Transcription Kit (Applied Biosystems). cDNAs were analyzed by real-time PCR using the Power SYBR Green PCR Master Mix (Applied Biosystems). Amplification, detection and data analysis were carried out with an ABI PRISM 7900HT Sequence Detection System and normalized to *GusB* or GAPDH expression. Changes in mRNA expression are noted as *x-*fold change relative to the control. qPCR primers are listed in Supplementary Table S7.

**References**

1. Lauden et al. Allogenicity of human cardiac stem/progenitor cells orchestrated by programmed death ligand 1. *Circ. Res.* **112**, 451-464 (2013).
2. Boukouaci et al. Natural killer cell crosstalk with allogeneic human cardiac-derived stem/progenitor cells controls persistence. *Cardiovasc. Res.* **104**, 290-302 (2014).
3. Moscoso et al. Podocalyxin-like protein 1 is a relevant marker for human c-kit+ cardiac stem cells. *J. Tissue Eng. Regen. Med.* **10**; 10.1002/term.1795 (2013).
4. Toran et al. Definition of human cardiac progenitor cell plasma membrane signature: comparative transcriptomics and proteomics analysis with human mesenchymal stem cells. *Stem Cell Reports*, under review.
5. [Martínez-Bartolomé](http://www.ncbi.nlm.nih.gov/pubmed/?term=Martínez-Bartolomé S%5BAuthor%5D&cauthor=true&cauthor_uid=18303013) et al. Properties of average score distributions of SEQUEST: the probability ratio method. [*Mol. Cell Proteomics*](http://www.ncbi.nlm.nih.gov/pubmed/?term=Martínez-Bartolome+and+2008) **7**, 1135-1145 (2008).
6. Bonzon-Kulichenko et al. [Revisiting peptide identification by high-accuracy mass spectrometry: problems associated with the use of narrow mass precursor windows.](http://www.ncbi.nlm.nih.gov/pubmed/25494653) *J. Proteome Res.* **14**, 700-710 (2015).
7. Navarro et al. General statistical framework for quantitative proteomics by stable isotope labeling, *J. Proteome Res.* **13**, 1234-1247 (2014).
8. Martin M. Cutadapt removes adapter sequences from high-throughput sequencing reads. EMBnet.journal **17**, 10-12 (2012)
9. Nakazato, T., Ohta, T., Bono, H. Experimental design-based functional mining and characterization of high-throughput sequencing data in the sequence read archive. PLoS ONE 8(10): e77910 (2013).
10. Li, B. and Dewey, C.N. RSEM: accurate transcript quantification from RNA-Seq data with or without a reference genome. B*MC Bioinformatics* **12**, 323 (2011).
11. Robinson, M.D., McCarthy, D.J. & Smyth, G.K. EdgeR: a Bioconductor package for differential expression analysis of digital gene expression data. *Bioinformatics* **26**, 139-140 (2010).
12. Alexa, A. and Rahnenfuhrer, J. topGO: Enrichment analysis for Gene Ontology. 2010, R package version 2.10.0. https://bioconductor.org/packages/release/bioc/html/topGO.html
13. Wencke, W., Sanchez-Cabo, F. & Ricote, M. GOplot: an R package for visually combining expression data with functional analysis. *Bioinformatics* **31**, 2912-2914 (2015).
14. [Laakmann](https://www.ncbi.nlm.nih.gov/pubmed/?term=Laakmann S%5BAuthor%5D&cauthor=true&cauthor_uid=23250337) et al. Minimally invasive closed-chest ultrasound-guided substance delivery into the pericardial space in mice. [*Naunyn Schmiedebergs Arch Pharmacol.*](https://www.ncbi.nlm.nih.gov/pubmed/23250337) **386**, 227-238 (2013).
15. Izarra et al. [miR-133a enhances the protective capacity of cardiac progenitors cells after myocardial infarction.](https://www.ncbi.nlm.nih.gov/pubmed/25465869) *Stem Cell Reports* **3**, 1029-42 (2014).

**Supplementary Figures**

**Supplementary figure legends**

**Figure S1. General scheme for CPC secretome definition. A.** Comparative selected FACS expression profile of CPC (purple) vs MSC (blue) and HDF (black); negative control (filled light blue areas). **B.** Scheme of the analysis used to define the specific CPC secretome.

**Figure S2. Description of the CPC secretome defined from conditioned medium.** LC-MS analysis defined a total of 754 proteins in Uniprot, after removing those common with CPC medium. After IPA analysis, 717 proteins were defined. Only 185 were defined specifically as belonging to the extracellular space compartment and associated to enzymatic activities. The remainder are associated to several subcellular compartments. The figure also shows the distribution of proteins (364) previously associated to the exosomal compartment and related to different subcellular compartments (<http://exocarta.org/index.html>).

**Figure S3. CPC secretome inferred from transcriptomics analysis.** **A.** Global differential expression profile of CPC secretome (682 proteins) compared with MSC/HDF, defined by mRNA-Seq. After IPA analysis, the CPC secretome was described based on canonical subcellular localization **(B)** or known function **(C)**. **D.** Analysis of differential expression profile (mRNA-Seq) of cytokines comparing CPC with MSC/HDF. **E.** Interaction network of the specific CPC secretome functions previously associated with angiogenesis (48 genes), indicating whether they are described in association with neovascularization, migration or cell movement of endothelial cells and overall, with angiogenesis. Genes are represented by nodes with their shape representing the type of molecule/functional class and the relationship between the nodes are indicated by edges. Nodes in red are upregulated and green color denotes downregulation. The intensity of colors indicates the degree of down- or upregulation, Edges connecting nodes are colored orange when leading to activation of the downstream node, blue when leading to its inhibition or yellow if the findings underlying the relationship are inconsistent with the state of the downstream node. Pointed arrowheads indicate that the downstream node is expected to be activated if the upstream node connected to it is activated, while blunt arrowheads indicate that the downstream node is expected to be inhibited if the upstream node that connects to it is activated

**Figure S4. Molecular and functional analysis of the combined CPC secretome**. **A.** Using the combined CPC secretome (546 proteins), main molecular and cellular functions were analyzed by IPA. **B.** Top 20 functional GO terms (p-values from 8.9 10^-7^ to 2.15 10^-4^) for growth factors identified in the combined CPC secretome include regulation of angiogenesis, cell migration and response to wounding and blood vessel morphogenesis.

**Figure S5. Role of CXCL6 in CPC conditioned medium. A.** Angiogenic activity in conditioned medium (CM) from the three CPC-1-3 isolates, compared with negative (C-) and positive controls (C+; VEGF, 5 ng/ml), using HUVEC (see Methods); bar, 500 μm. After 6 h, images were acquired and analyzed using ImageJ. The assay was performed three times and data are expressed as mean ± SD; black lines summarize p‑values (***<0.001, **<0.01, *<0.05, ns = not significant) for CPC vs. negative control (one-way analysis of variance followed by Bonferroni correction for multiple comparison). **B.** Migratory activity of CPC CM on THP-1 cells and migration index was calculated for the CPC isolates (CPC-1-3), compared to CPC culture medium alone, recombinant CXCL6 (10 mM) and negative control. **C, D.** Titration of anti-CXCL6 antibody. THP-1 cells were challenged with recombinant CXCL6 (1 or 10 nM; +) and incubated with increasing amounts of anti-CXCL6 (0, 10, 50, 100 nM). **D.** Inhibition of CXCL6-induced migration (3 h) in THP-1 cells by anti-CXCR1 or -CXCR2 (5 nM) antibodies; migrated cells were counted in BD TruCOUNT tubes and the chemotaxis index established. Data are expressed as mean ± SD, *n* = 3; black lines summarize p‑values (***<0.001, **<0.01, *<0.05, ns = not significant) of CXCL6 vs. anti-CXCL6 or anti-CXCR1/CXCR2 (one-way analysis of variance followed by Bonferroni correction for multiple comparison). **E.** Comparative migration response of MSC and CPC to different CXCL6 concentrations (1, 10, 100 nM).

**Supplementary Figures**


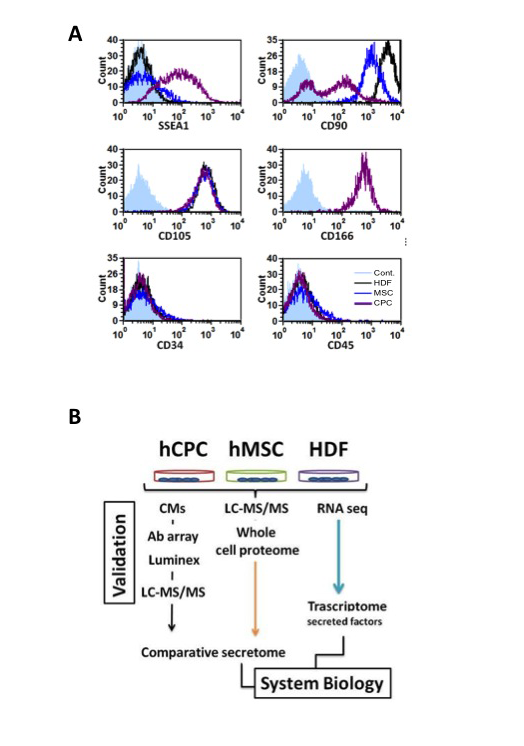


**Figure S1. General scheme for CPC secretome definition.**

**
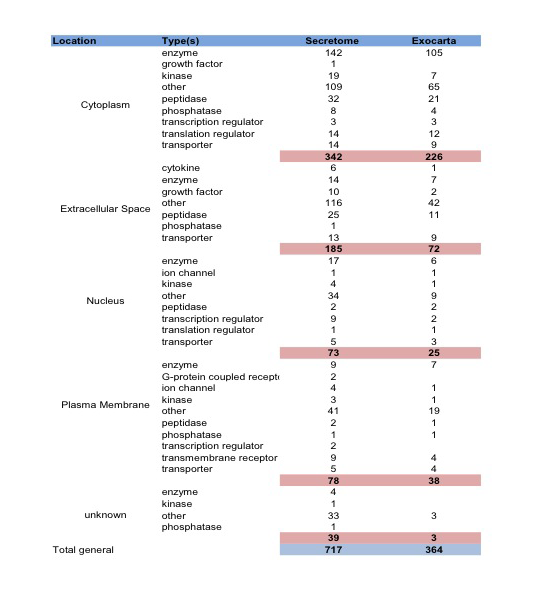
**

**Figure S2. Description of the CPC secretome, defined from conditioned medium.**

**Figure S3. CPC secretome inferred from transcriptomics analysis.**


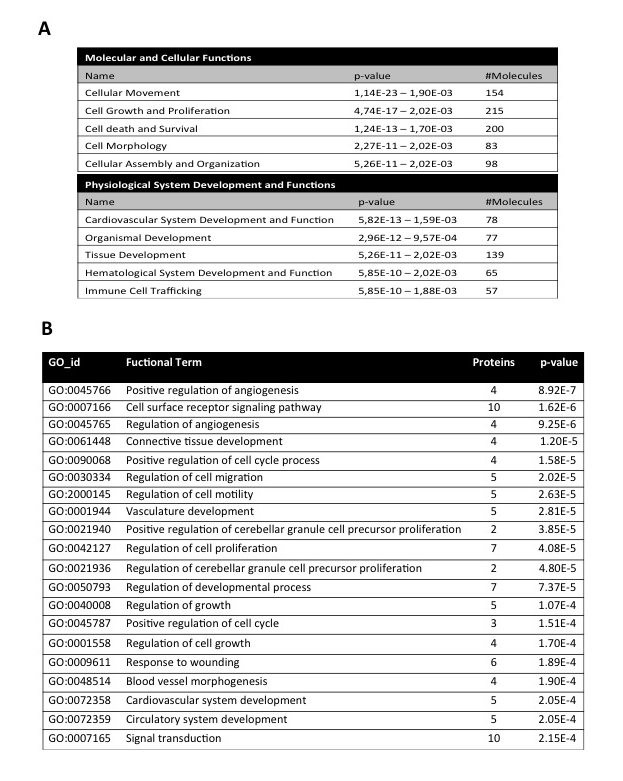


**Figure S4. Molecular and functional analysis of the combined CPC secretome**

**
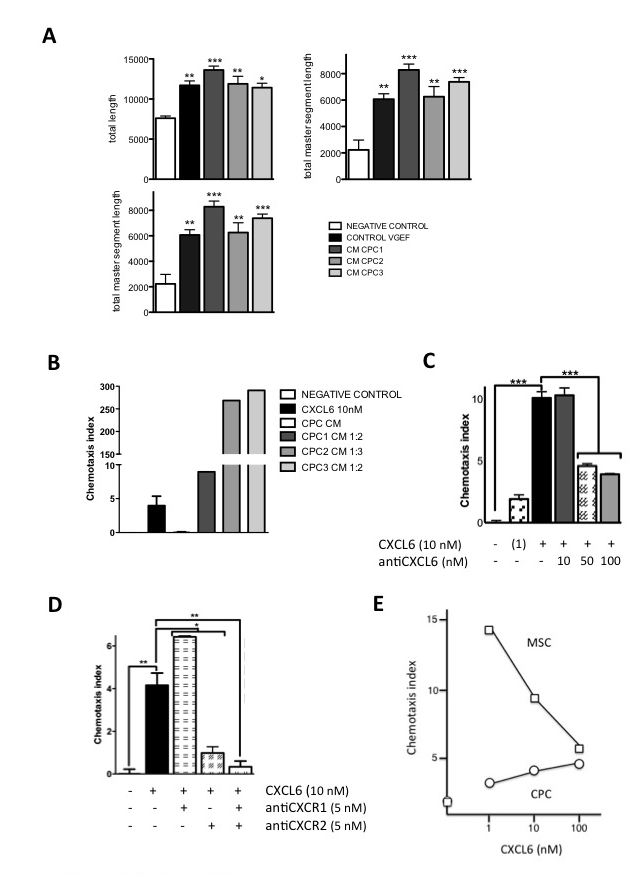
**

**Figure S5. Role of CXCL6 in CPC conditioned medium**

**Supplementary Tables**

**Table legends**

**Table S1.** CPC secretome defined from whole label-free proteome. (xlsx)

**Table S2.** Comparative analysis of the CPC secretome (conditioned medium) with MSC & HDF. The complete list (185 genes) shows those that have been previously associated with exosomes in yellow. (xlsx)

**Table S3.** Comparative validation analyses of the CPC secretome. (pdf)

**Table S4.** Extracellular proteins of CPC inferred from transcriptional analysis. (pdf)

**Table S5.** Comparative expression analysis in CPC *vs.* MSC/HFD of the *adhesion* category. Genes significantly overexpressed (> 5-LogFC) or downregulated (< 5-LogFC) in CPC with respect to MSC/HFD are indicated in yellow and green, respectively. Those genes similarly regulated in CPC vs. MSC/HFD are framed and those most clearly regulated are indicated in bold. (pdf)

**Table S6.** Antibodies used in the study.

**Table S7.** Primers used in the study.

**Tables S1, S2, S4 & S5.** *Submitted as independent files (xlsx).*

**
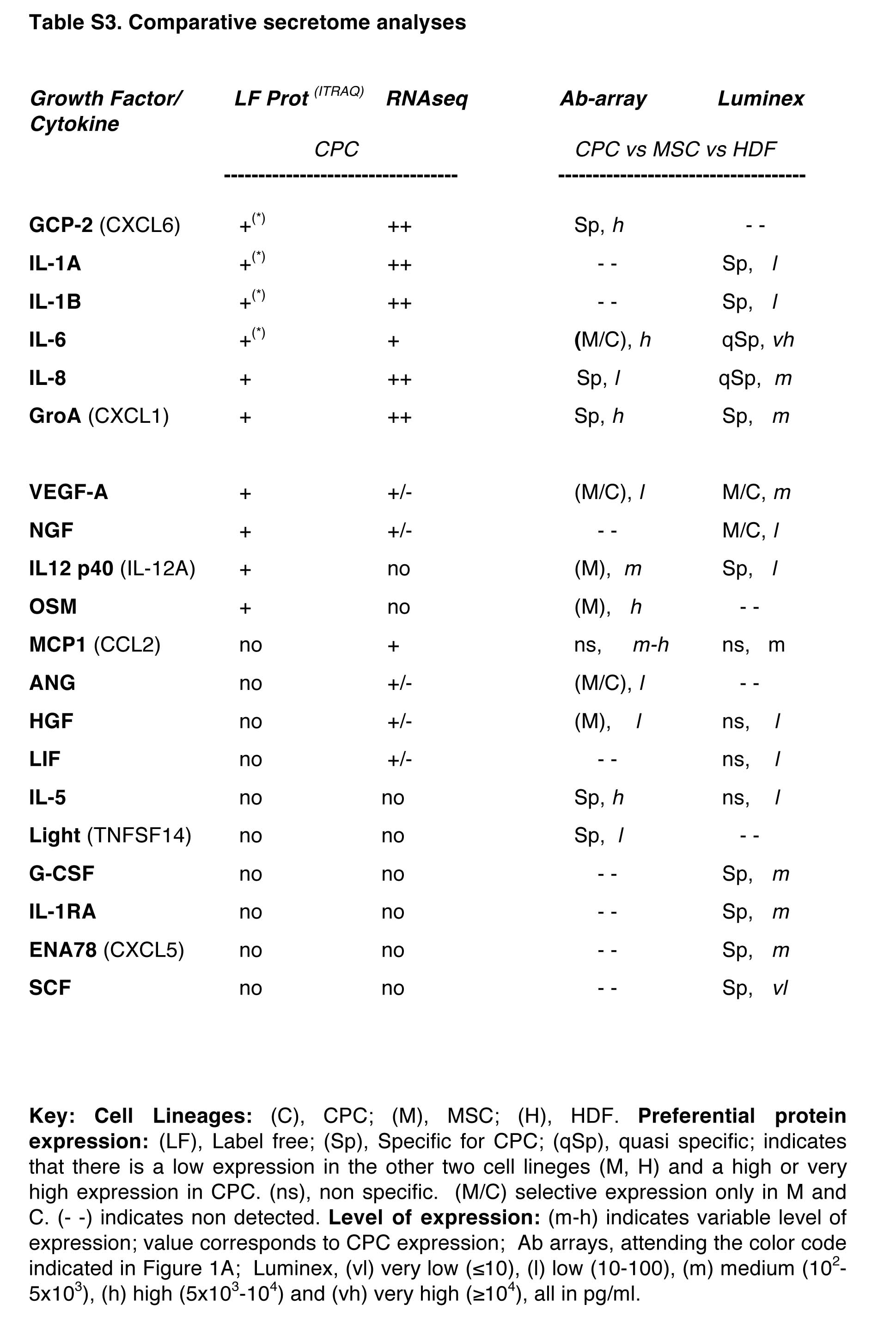
**

**Table S6.** Antibodies used in the study

| Primary Antibodies | Reference | Source |
| --- | --- | --- |
| anti-hu m an Stage-Specific Embryonic  Antigen-1 (SSEA-1); mouse. | MAB-4301 | Chemicon, Temecula, CA |
| a nt i - h um a n C D 9 0 , FI T C c o n j u g a t e ;  mouse. | 555595 | BD Pharmingen, Heidelberg, Germany |
| a nt i - h um a n CD 1 0 5 / E n d o g l i n ;  FI T C co n j u g a t e ; mouse. | MA1-80943 | Thermo Scientific, Waltham, MA |
| anti-CD166 (ALCAM); mouse. | 559260 | BD Pharmingen |
| a nt i - h um a n C D 3 4 , PE co n j u g a t e ;  mouse. | 555822 | BD Pharmingen |
| ant i - hu m an C D 45 , F IT C  co n j u g a t e ; mouse. | 345808 | BD Pharmingen |
| ant i - human GCP-2 (T-16); go a t . | Sc-5813 | Santa Cruz Biotechnology, Santa Cruz, CA |
| ant i - human CXCR1/IL8 RA; mouse. | MAB330 | R&D Systems, Minneapolis, MN |
| ant i - human CXCR2/IL8 RB; mouse. | MAB331 | R&D Systems |
| anti-human laminin; ra b b i t . | Ab11575 | Abcam, Cambridge, MA |
| anti- human beta actin; mo u s e . | Ab8226 | Abcam |
| anti-human IGFIR; ra b b i t . | (111A9) 3018 | Cell Signaling Technology, Danvers, MA |
| anti-hu m an Met (c-MET); ra b b i t . | Ab39075 | Abcam |

| Secondary Antibodies | Reference | Source |
| --- | --- | --- |
| Goat Anti-rabbit HRP co n j u g a t e | P0448 | Dako, Glostrup Denmark |
| Rabbit Anti-Mouse HRP co n j u g a t e | P0260 | Dako, Glostrup Denmark |
| Rabbit Anti-Goat HRP co n j u g a t e | P0449 | Dako, Glostrup Denmark |
| Do n k e y a n ti - Ra b b i t I g G ( H + L ) ; Al e x a F l u o r ® 4 8 8 c o n j u g a t e | A-21206 | Thermo Scientific, Waltham, MA |
| Go a t ant i - Ra b b i t I g G (H + L); Al e x a  Fl u o r ® 5 4 6 c o n j u g a t e | A-11035 | Thermo Scientific, Waltham, MA |
| Goat anti-Mouse IgG (H+L); Alexa Fluor®  546 conjugate | A-11030 | Thermo Scientific, Waltham, MA |
| Ra t A n t i - Mo u s e I g M , P E  co n j u g a t e | 553409 | BD Pharmingen, Heidelberg, Germany |
| Ra t An t i - Mo u s e I g G 1 , P E  co n j u g a t e | 340270 | BD Pharmingen, Heidelberg, Germany |

**Table S7.** Primers used in the study.

| **Gene** | **Sequence 5´-3´** |
| --- | --- |
| *Ctgf* | Fw: GCTTGGCGATTTTAGGTGTC  Rv: CAGACTGGAGAAGCAGAGCC |
| Desmin (*Des*) | Fw: TACACCTGCCAGATTGATGC  Rv: ACATCCAAGGGCATGTTCAC |
| *GusB* | Fw: ACTCCTCACTGAACATGCGA  Rv: ATAAGACGCATCAGAAGCCG |
| *Gadph* | Fw: AGGTCGGTGTGAACGGATTTG  Rv: TGTAGACCATGTAGTTGAGGTCA |
| *Bnp* | Fw: AGTCCTTCGGTCTCAAGGCA  Rv: CCGATCCGGTCTATCTTGTGC |
| *Actc 1* | Fw:GCTCTGGGCTTCATCACCTA  Rw:AGCTGTCTTCCCGTCCATC |
| *Col-Iα* | Fw: TGACTGGAAGAGCGGAGAGT  Rv: GTTCGGGCTGATGTACCAGT |
| *αSMA* | Fw: CTGACAGAGGCACCACTGAA  Rv: CATCTCCAGAGTCCAGCACA |
